# Stage-Associated Plant–Soil–Microbiome Patterns and Rhizosphere Pb–Zn Stabilization in *Indocalamus decorus* at a Mining Site

**DOI:** 10.3390/toxics14100897

**Published:** 2026-10-10

**Authors:** Zhen Miao, Fulin Yuan, Qiyuan Zou, Chenyanan Yuan, Yi Zheng, Kexin Wang, Bingyang Lyu, Zhenghua Luo, Mingyan Jiang

**Affiliations:** 1 College of Landscape Architecture, Sichuan Agricultural University, Chengdu 611130, China; alvs01@163.com (Z.M.);; 2 Chengdu Botanical Garden, Chengdu 610083, China

**Keywords:** lead, zinc, metal bioavailability, phytostabilization, rhizosphere microbiome, *Indocalamus decorus*

## Abstract

*Indocalamus decorus* is a perennial bamboo with potential for phytomanagement, but its Pb–Zn partitioning and associated microbiome under field co-contamination remain unclear. Parallel plantings were established at a Pb–Zn mining site and the provenance site. Plant nutrients, rhizosphere properties, Pb–Zn concentrations and soil fractions, and bacterial and fungal communities were evaluated across four developmental stages at the mining site; shoot initiation was also evaluated at the provenance site. At shoot initiation, mine-site plants had lower organ P concentrations, whereas root N was maintained. Rhizosphere nutrient availability was relatively high during shooting. Pb accumulated mainly in roots, while Zn was transferred more readily to culms; organ-specific bioconcentration factors for both metals remained below 1. Compared with bulk soil, rhizosphere soil contained lower DTPA-extractable Pb and Zn, smaller acid-soluble/exchangeable fractions, and larger residual fractions. Sample compartment explained 60.8% and 66.9% of bacterial and fungal community variation, respectively. Rhizosphere fungi were associated with a broader range of metal fractions and plant metal-distribution indices than other microbial compartments. These results indicate root-dominated Pb retention, reduced rhizosphere Pb–Zn lability, and compartment-specific microbial associations, supporting further evaluation of *I. decorus* as a candidate for phytostabilization-oriented management of Pb–Zn-contaminated mine soils.

## 1. Introduction

Metal contamination of mine tailings and adjacent soils restricts soil functioning and vegetation establishment. Pb is toxic and environmentally persistent [1]. Although Zn is essential for plants, excessive Zn can inhibit plant growth and disrupt physiological processes. Their co-occurrence can reduce soil fertility and delay ecosystem recovery [2,3]. Plant-based management can stabilize contaminated surfaces with relatively little disturbance, but conventional hyperaccumulators often have low biomass, slow growth, and limited field adaptability [4]. High-biomass perennial plants that maintain persistent cover and belowground growth may therefore be more suitable for the long-term management of contaminated sites. Their phytomanagement potential should be evaluated by combining plant growth and nutrient status with metal accumulation, translocation, availability, and soil fractionation.

Bamboos are perennial woody grasses with rapid vegetative growth, extensive rhizome systems, repeated shoot regeneration, and high biomass production, making them potential candidates for phytomanagement of metal-contaminated soils [5]. Their clonal growth may facilitate establishment across spatially heterogeneous contaminated soils [6]. Persistent root, rhizome, and canopy cover may also reduce soil erosion and the dispersal of contaminated particles [7]. *Indocalamus decorus* is an evergreen shrubby bamboo with dense culms and vigorous belowground growth. Previous research showed that it maintained a relatively high root-to-shoot ratio and substantial C storage under adverse soil conditions [8]. Mowing has been examined as a way to regulate biomass and metal removal, and chelator application has also been tested to enhance metal mobilization and uptake [9,10]. However, the growth performance of *I. decorus* in Pb–Zn-contaminated mine soils and the associated rhizosphere metal availability and fractionation remain poorly understood.

Plant-associated microorganisms can influence nutrient acquisition, stress tolerance, and metal transformation and may therefore contribute to phytomanagement [11]. Rhizosphere microorganisms interact directly with soil minerals, metal ions, and root-derived compounds, whereas microorganisms within surface-sterilized plant tissues occupy the more strongly host-filtered endosphere. Cultured endophytes demonstrate the potential importance of these interactions. *Sphingomonas* sp. ZYG-4 isolated from *Ageratina adenophora* roots increased plant biomass and Pb uptake [12]. *Aspergillus luchuensis* C7 isolated from Prosopis laevigata roots enhanced photosynthesis and Pb accumulation through changes in glutathione metabolism [13]. Microorganisms can also transform or immobilize Pb through biosorption and the formation of phosphate-, sulfide-, or carbonate-containing minerals [14]. Microbiome management is consequently expanding from individual strains to synthetic communities and the manipulation of resident microbial assemblages [12]. Nevertheless, the rhizosphere and endosphere microbiomes of perennial plants under field Pb–Zn contamination remain insufficiently characterized, particularly across plant developmental stages.

To address these gaps, parallel plantings of *I. decorus* were established at a Pb–Zn mining site and its provenance site in January 2022. Site-associated differences were evaluated at shoot initiation, and stage-associated variation was examined across four sampling stages at the mining site. We integrated plant biomass and nutrient status, rhizosphere properties, Pb–Zn concentrations and soil fractions, and bacterial and fungal communities. We hypothesized that (1) mine-site conditions would be associated with altered plant growth, nutrient concentrations, and organ-specific Pb–Zn distribution, together with lower rhizosphere metal availability; (2) microbial communities would differ among ecological niches and sampling stages; and (3) rhizosphere and endosphere communities would show distinct associations with plant nutrition and metal distribution. This study aimed to evaluate the phytostabilization potential of *I. decorus* and characterize associations among plant nutrients, soil metal fractions, and microbial communities under field Pb–Zn contamination.

## 2. Materials and Methods

### 2.1. Experimental Site and Plant Materials

**Figure 1:**
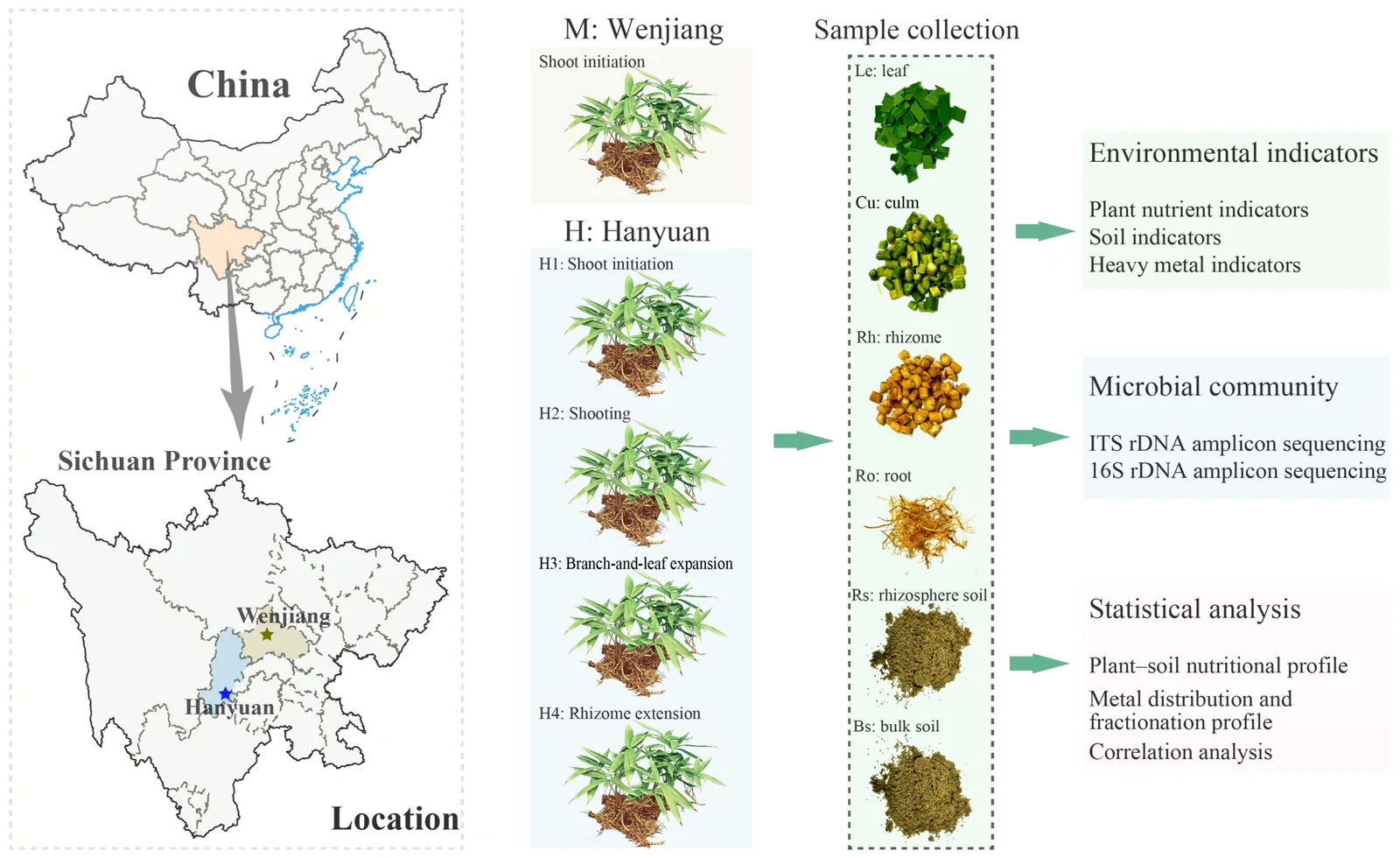
Study sites, sampling design, and analytical workflow. M, shoot initiation at the provenance site in Wenjiang; H1–H4, shoot initiation, shooting, branch-and-leaf expansion, and rhizome extension at the mining site in Hanyuan, respectively. Bs, bulk soil; Rs, rhizosphere soil; Ro, roots; Rh, rhizomes; Cu, culms; Le, leaves. These abbreviations apply throughout subsequent figures and tables.

Plant material of *I. decorus* was obtained from the living collection at the Bamboo Resource Garden of Sichuan Agricultural University, Chengdu, China. Species identity was confirmed morphologically, using voucher specimen PE 01512448 as a taxonomic reference. Field experiments were conducted at two sites: a lead–zinc mining site in Sichuan Province, Hanyuan County (Site H: contaminated soil; 29°22′ N, 102°40′ E; 951.9 m a.s.l.), and the provenance site, a bamboo germplasm garden in Wenjiang District, Chengdu (Site M: provenance soil; 30°42′ N, 103°51′ E; 530 m a.s.l.; Figure 1). Site H has a mean annual temperature of 17.9 °C and mean annual precipitation of 741.8 mm. The tailing soil is primarily contaminated with Pb (1285.42 ± 73.16 mg kg^−1^) and Zn (1045.83 ± 52.08 mg kg^−1^), is slightly alkaline, and exhibits low fertility (Table 1). Site M has a mean annual temperature of 16.8 °C and mean annual precipitation of 960 mm. Its soil is also slightly alkaline but more fertile (Table 1).

Planting occurred in January 2022. Prior to planting, both sites were cleared of surface weeds and debris. At Site H, a ~5 cm layer of clean exogenous soil (Table 1) was uniformly applied to the surface. Both sites were then plowed to ~20 cm depth, with Site H plowed repeatedly to ensure thorough mixing of the exogenous and contaminated soils. One-year-old *I. decorus* ramets from Site M were used; each clump was pruned to eight culms, and 20 clumps were planted per site. Only essential irrigation and weeding were applied during the growth period.

### 2.2. Sample Collection and Processing

Samples were collected from October 2024 to July 2025 at four developmental stages of *I. decorus*: shoot initiation (October 2024), shooting (January 2025), branch-and-leaf expansion (April 2025), and rhizome extension (July 2025). Shoot initiation was sampled at both Sites H and M; the remaining three stages were sampled only at Site H. At each site–stage sampling event, six clumps exhibiting relatively uniform growth were randomly selected from the 20 established clumps and treated as six independent biological replicates. Rhizosphere soil (Rs), defined as soil adhering closely to the root surface after excavation, and roots (Ro), rhizomes (Rh), culms (Cu), and leaves (Le) were collected separately from each selected clump. Bulk soil (Bs) was collected from six independent sampling points located at least 2 m from any plant, with each sampling point representing one biological replicate (*n* = 6). At Site H, clump selection was conducted independently at each developmental stage; therefore, the sampled clumps could differ among stages. All samples were transported on ice and processed within 24 h. Soil samples were cleared of debris, and plant tissues were rinsed, blotted dry, and cut into approximately 5-mm pieces. A total of 180 individual samples were used for nutrient, soil-property, enzyme-activity, and metal analyses, with six within-site biological replicates per sample type at each site–stage combination (*n* = 6). Biomass was assessed separately by destructive sampling of three clumps per group at M, H1, and H4 (*n* = 3). Ro, Rh, Cu, and Le were separated for dry-mass determination. For amplicon sequencing, aliquots from the six samples of each sample type were randomly pooled in pairs to form three composite biological replicates, yielding 90 sequencing samples in total (n = 3; Figure 1).

### 2.3. Determination of Plant Nutrients, Soil Properties, and Enzyme Activities

Plant organ samples were heated at 105 °C for 30 min and then oven-dried at 80 °C to constant weight, ground, and passed through a 0.25-mm sieve. Soil samples were air-dried and passed through a 1-mm sieve. Soil physicochemical properties were determined following the methods of Klesta and Bartz [15], and plant nutrient indices were analyzed according to Ge and Xie [16]. Briefly, total carbon (TC) and soil organic matter (SOM) in soil, and TC in plant samples, were measured using the K_2_Cr_2_O_7_–H_2_SO_4_ external heating oxidation method. Total nitrogen (TN) and total phosphorus (TP) were determined after H_2_SO_4_–HClO_4_ digestion using the Kjeldahl method and the vanadate-molybdate yellow colorimetric method, respectively. Soil pH was measured potentiometrically at a soil-to-water ratio of 1:2.5 (*w*/*v*). Available nitrogen (AN) was determined using the alkaline diffusion method, and available phosphorus (AP) was extracted with 0.5 mol·L^−1^ NaHCO_3_ (Olsen-P) and quantified by the molybdenum blue ascorbic acid colorimetric method.

Soil enzyme activities, including urease, sucrase, β-glucosidase (β-Gase), fluorescein diacetate hydrolysis (FDAase), and neutral phosphatase (Pase), were determined using enzyme-specific substrates and buffers. General methodological guidance was provided by Dick [17]. For each assay, 5 g of air-dried soil passed through a 1-mm sieve was incubated at 37 °C for 1 h. Urease activity was determined using 10 mL of 10% urea solution and citrate buffer (pH 6.7). The ammonium released was quantified by phenol–sodium hypochlorite colorimetry at 578 nm [18]. Sucrase activity was determined using 15 mL of 8% sucrose solution and phosphate buffer (pH 5.5). The reducing sugars released were quantified by 3,5-dinitrosalicylic acid (DNS) colorimetry at 508 nm [19]. β-Gase activity was determined using 10 mL of 1% salicin solution and modified universal buffer (MUB, pH 6.0). The glucose released was quantified by DNS colorimetry at 508 nm. FDAase was determined using 5 mL of 4.9 mM fluorescein diacetate solution and phosphate buffer (pH 7.6). The fluorescein released was measured at 490 nm [20]. Pase activity was determined using 5 mL of 50 mM p-nitrophenyl phosphate (pNPP) solution and MUB (pH 6.7). The p-nitrophenol (pNP) released was measured at 400 nm [21].

### 2.4. Determination of Pb and Zn Concentrations and Fractionation

Air-dried soil samples and oven-dried plant-organ samples from Site H were ground and passed through a 0.25-mm sieve. Following the digestion method described in Gao et al. [22], 0.2 g of each sample was digested with 5 mL of HNO_3_ and 1 mL of HClO_4_. After digestion, the digest was diluted to a final volume of 50 mL with 2% (*v*/*v*) HNO_3_. Pb and Zn concentrations were determined by inductively coupled plasma optical emission spectrometry (ICP-OES; PerkinElmer Optima 8000, PerkinElmer, Waltham, MA, USA). Calibration curves were prepared using Pb (GSB 04-1742-2004) and Zn (GSB 04-1761-2004) standard solutions supplied by Guobiao (Beijing) Testing and Certification Co., Ltd., China. Analytical quality control included spike-recovery tests, with standards added to the samples before digestion. Mean spike recoveries were 95.72 ± 2.25% for Pb and 96.87 ± 3.27% for Zn (mean ± SD, n = 3). The detection limits in the measured solutions were 1.5 µg L^−1^ for Pb and 0.5 µg L^−1^ for Zn. The bioconcentration factor (BCF) and translocation factor (TF) were calculated as follows:

BCF_X_ = C_X_/C_Rs_,

where X represents Ro, Rh, Cu, or Le. Total metal concentrations in rhizosphere soil were used to relate tissue concentrations to those in the soil adhering to the sampled roots. These rhizosphere-referenced BCFs are not directly interchangeable with values calculated using bulk or pre-planting soil.

TF_X/Y_ = C_X_/C_Y_,

where X and Y represent Ro, Rh, Cu, or Le.

Following Rauret et al. [23], Pb and Zn in bulk and rhizosphere soils were fractionated by BCR sequential extraction into F1 acid-soluble/exchangeable, F2 reducible, F3 oxidizable, and F4 residual fractions. DTPA-extractable metals were used as a proxy for potential bioavailability, following Xiao et al. [24] and Yang et al. [10]. Briefly, 10 g soil was shaken with 20 mL DTPA extractant (0.005 M DTPA + 0.01 M CaCl_2_ + 0.1 M TEA, pH 7.3) for 2 h and centrifuged. A 5 mL supernatant aliquot was digested with 5 mL concentrated HNO_3_ at 170 °C to near dryness and diluted to 50 mL. Pb and Zn in extracts were measured by ICP-OES. BCR fraction concentrations were expressed as mg kg^−1^ soil and as percentages of the summed concentrations of F1–F4. DTPA-extractable Pb and Zn concentrations were expressed as mg kg^−1^ soil. BCR fraction contents and recoveries for Pb and Zn are shown in Tables S1 and S2, respectively.

### 2.5. Amplicon Sequencing, Data Processing, and Analysis

Before DNA extraction, soil samples were passed through a 2-mm sieve, and plant samples were surface-sterilized following Miao et al. [8]. To verify sterilization effectiveness, 100 µL of the final rinse water was spread on TSA medium (Hopebio, Qingdao, China; HB0177-1) and incubated at 28 °C in the dark for 5 days. After no microbial growth was observed, samples were stored at −80 °C. The fungal ITS1 region was amplified using the ITS1-1F/ITS1-1R primer pair, and the V3–V4 region of the bacterial 16S rRNA gene was amplified using primers 341F/806R on a Bio-Rad T100 thermal cycler. High-throughput sequencing was performed by Novogene Co., Ltd. (Beijing, China). Sequence processing and taxonomic analysis followed the pipeline described by Miao et al. [8].

### 2.6. Statistical Analysis

Data were processed in Microsoft Excel 2021. Univariate analyses and graphing were performed in GraphPad Prism 10.1.2. Comparisons between M and H1 were interpreted as site-associated differences at the shoot-initiation stage, whereas comparisons among H1–H4 were interpreted as stage-associated differences within Site H. Group differences were assessed using one-way ANOVA followed by Tukey’s HSD test at *p* < 0.05. Cluster heatmaps (ComplexHeatmap 2.12.0), Venn diagrams (VennDiagram 1.6.20), correlation analyses (stats), t-distributed stochastic neighbor embedding (t-SNE, Rtsne 0.17) [25], and principal coordinate analysis (PCoA; phyloseq 1.40.0) were generated in R 4.3.2 [26]. Genus-level differences between M and H1 were screened separately for rhizosphere and endosphere via OPLS-DA. Genera with Welch’s *p* < 0.05, VIP > 1, and FC (H1/M) > 2 or <0.5 were considered differential and visualized by volcano plots; positive/negative log_2_FC indicates higher abundance in H1/M. Permutational multivariate analysis of variance was used to evaluate community differences based on Bray–Curtis dissimilarities. PCA was used for exploratory visualization, and OPLS-DA was implemented in MetaboAnalystR to identify discriminatory variables. Model performance was evaluated using seven-fold cross-validation and 200 permutation tests; variables with VIP > 1 were retained as key discriminatory indicators. Comprehensive scores were calculated by integrating VIP-weighted variables using a membership-function approach [27]. Mantel tests (ggcor 0.9.8.1) were used to examine associations among selected plant, rhizosphere, heavy-metal, and microbial-community variables [28]. For Mantel analyses, physicochemical measurements from the two clumps contributing to each composite microbiome sample were averaged, yielding three matched observations per group.

## 3. Results

### 3.1. Plant, Soil, and Metal Responses

#### 3.1.1. Plant Nutrient Responses

**Figure 2:**
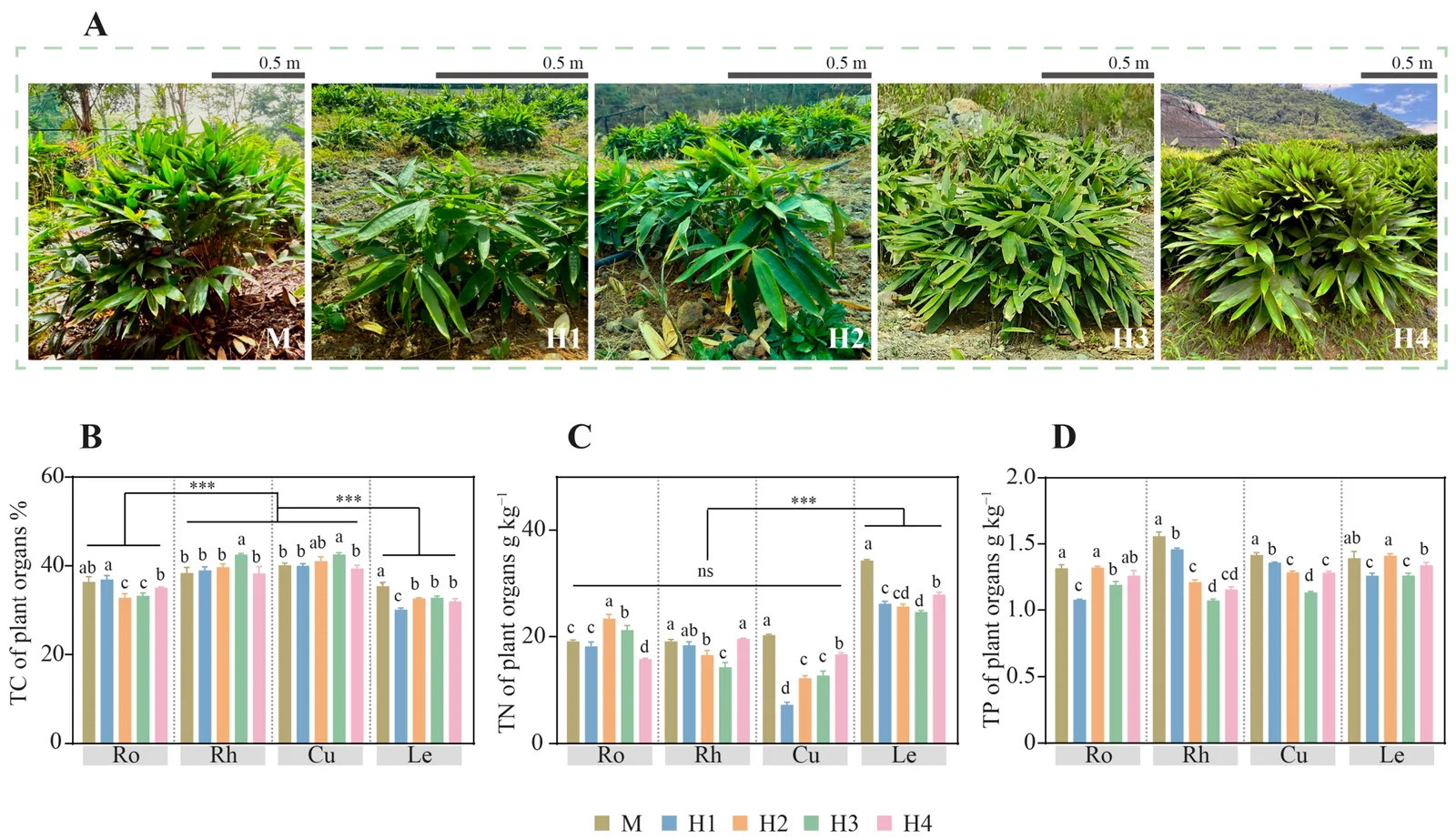
**Plant nutrient concentrations across sampling groups.** (**A**) Representative plants; scale bars = 0.5 m. (**B**–**D**) Total carbon (TC), total nitrogen (TN), and total phosphorus (TP), respectively. Bars show means ± SD (*n* = 6). Different lowercase letters indicate differences among sampling groups within each organ (one-way ANOVA with Tukey’s HSD, *p* < 0.05). Brackets compare the indicated compartments using observations pooled across sampling groups. ns, *p* ≥ 0.05; ***, *p* < 0.001. Statistical notation applies to subsequent figures unless otherwise specified.

Representative phenotypes and biomass measurements indicated differences in plant performance between the provenance and mining sites and across the sampling sequence (Figure 2A and Figure S1F). At shoot initiation, total biomass was approximately 0.81 kg clump^−1^ at Site M and 0.47 kg clump^−1^ at H1. Within Site H, total biomass reached approximately 1.12 kg clump^−1^ at H4, with leaves, culms, and rhizomes accounting for most of the higher biomass relative to H1. Because biomass was quantified only at M, H1, and H4, these values represent same-stage site-associated differences and the difference between the initial and final sampling stages at Site H, respectively. Plant C, N, and P concentrations differed among organs and sampling groups (Figure 2B–D). At shoot initiation, culm and leaf TN concentrations were lower at H1 than at M, whereas root TN was maintained. Leaf TC was also lower at H1, while TP concentrations were generally lower across plant organs at H1 than at M. Within Site H, root TN was relatively high at H2 and H3, whereas rhizome TN was relatively high at H1 and H4 and culm TN was highest at H4. Rhizome, culm, and leaf TC concentrations were relatively high at H2 and H3, while root TC showed an opposing pattern. Rhizome and culm TP concentrations generally decreased from H1 to H3 and increased again at H4, whereas root and leaf TP concentrations were relatively high at H2 and H4.

#### 3.1.2. Rhizosphere Soil Properties and Enzyme Activities

**Figure 3:**
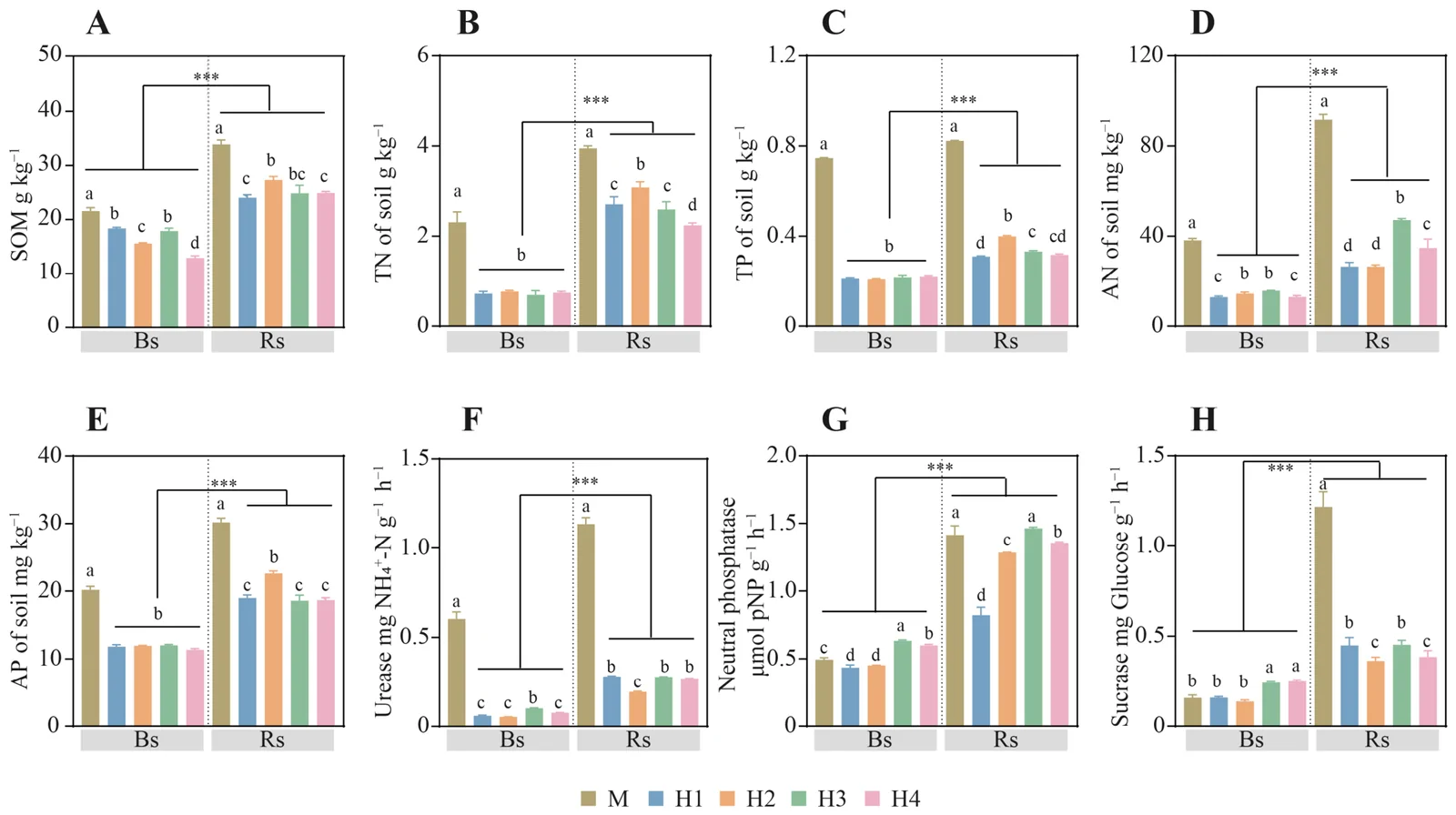
**Soil nutrients and enzyme activities across sampling groups.** (**A**–**E**) SOM, TN, TP, AN, and AP, respectively. (**F**–**H**) Urease, neutral phosphatase (Pase), and sucrase activities, respectively. Bars show means ± SD (*n* = 6). Letters compare sampling groups within each soil compartment; brackets compare pooled observations between compartments. ***, *p* < 0.001.

Rhizosphere soil generally contained higher nutrient concentrations than bulk soil, whereas soil pH remained slightly alkaline across all sampling groups (Figure 3A–E and Figure S1A). At shoot initiation, SOM, TN, TP, AN, and AP concentrations were lower at H1 than at M in both soil compartments. Within Site H, nutrient variation was more pronounced in rhizosphere than in bulk soil. Rhizosphere SOM, TN, TP, and AP were relatively high at H2, whereas AN was highest at H3. Most of these nutrient indices subsequently declined at H4, although their values generally remained higher in rhizosphere than in bulk soil. Soil enzyme activities showed enzyme- and stage-specific patterns (Figure 3F–H and Figure S1B,C). At shoot initiation, urease and FDAase activities in bulk soil and urease, sucrase, β-glucosidase, and FDAase activities in rhizosphere soil were lower at H1 than at M, whereas bulk-soil β-glucosidase showed the opposite pattern. Across H1–H4, rhizosphere Pase activity was highest at H3, while FDAase activity was lowest at this stage. Rhizosphere urease and sucrase activities were relatively high at H1 and H3, whereas β-glucosidase activity was lowest at H2 and increased at H3 and H4.

#### 3.1.3. Pb and Zn Distribution, Translocation, and Rhizosphere Fractionation

**Figure 4:**
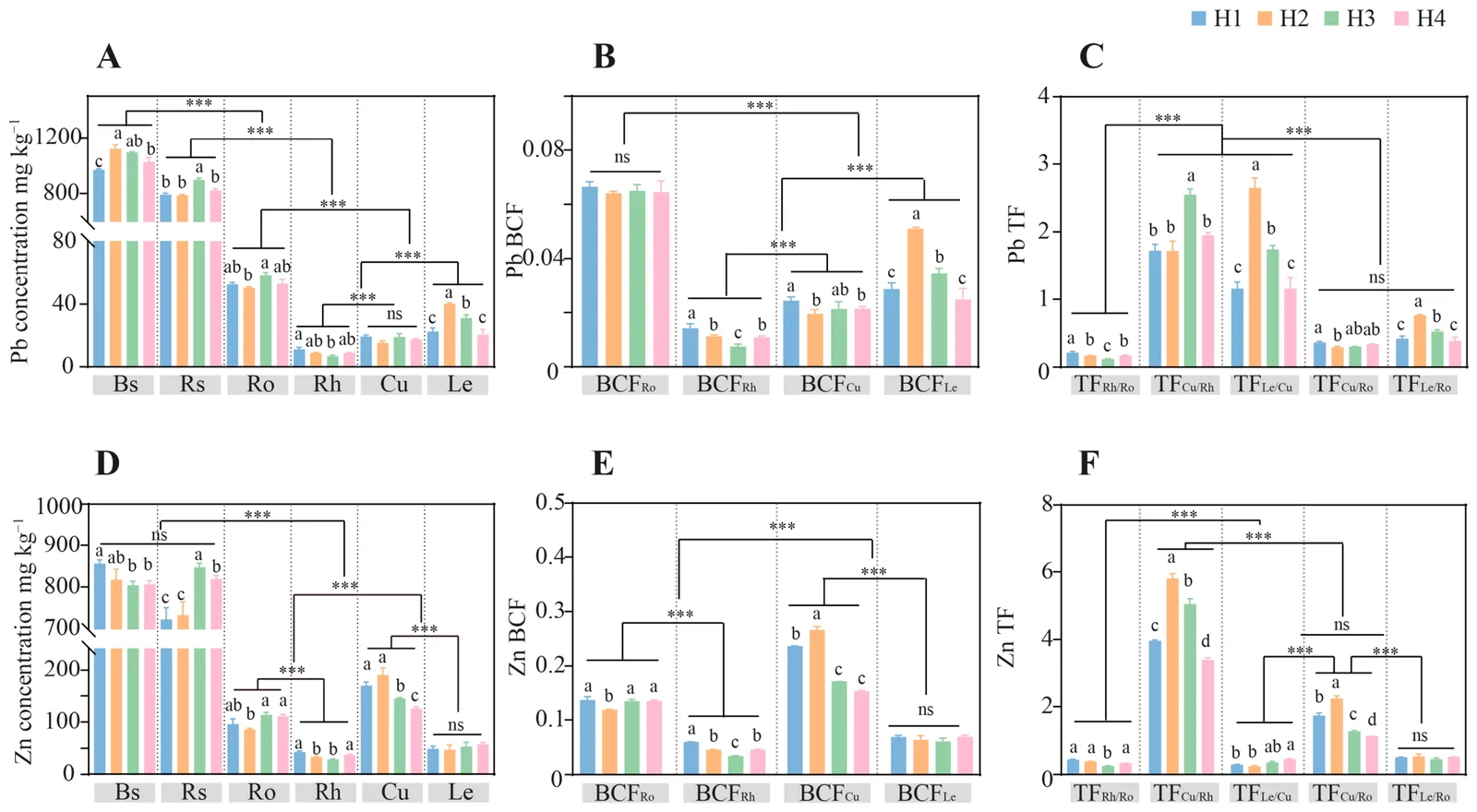
**Pb and Zn concentrations and concentration ratios at Site H.** (**A**–**C**) Pb concentrations, bioconcentration factors (BCFs), and translocation factors (TFs), respectively. (**D**–**F**) Corresponding Zn measurements. BCF_x_ = organ x concentration/rhizosphere-soil concentration; TF_x_/_γ_ = organ x concentration/organ y concentration. Bars show means ± SD (*n* = 6). Letters compare stages within each compartment or indicator; brackets compare pooled observations between the indicated compartments or indicators. ***, *p* < 0.001.

**Figure 5:**
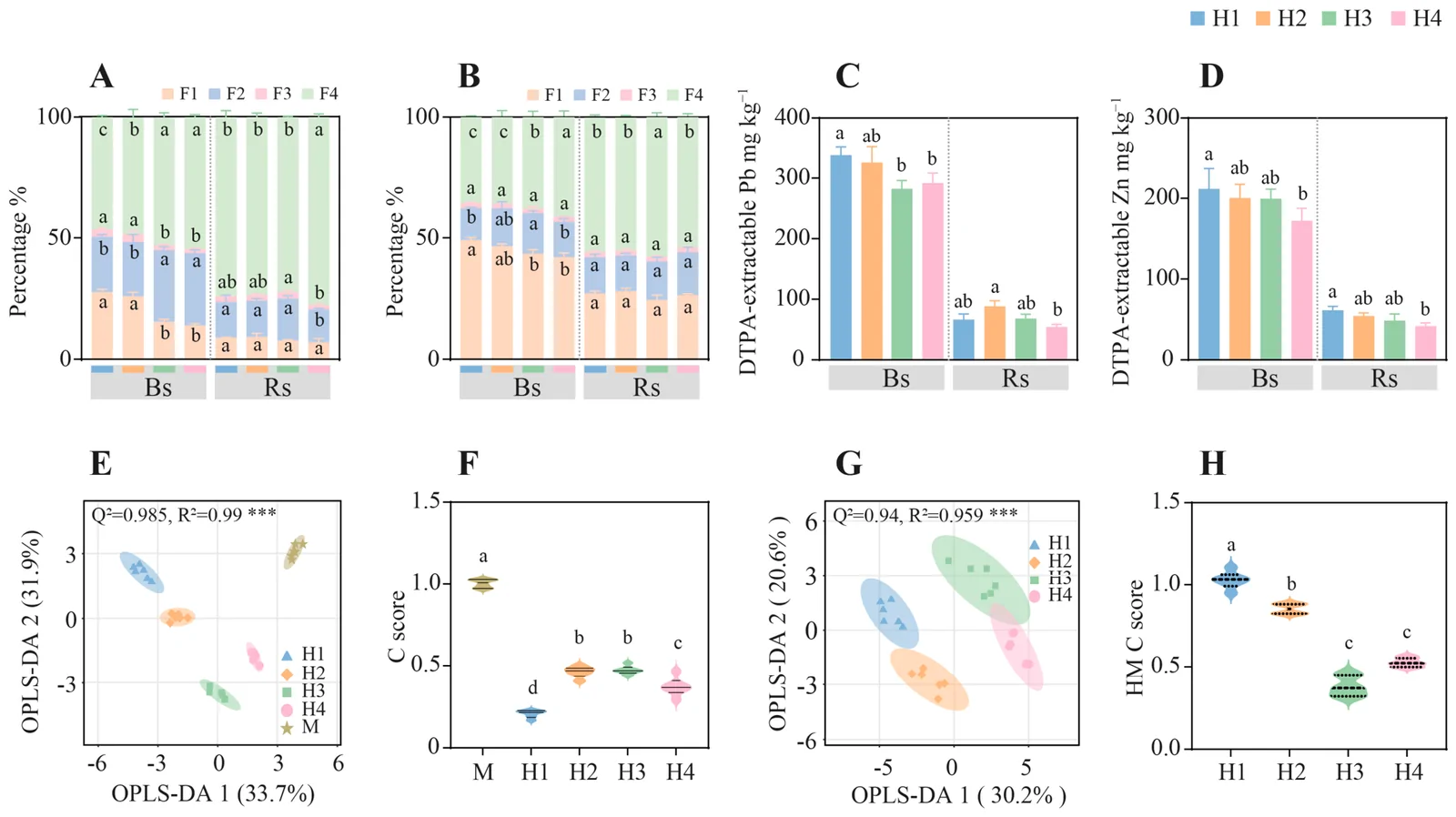
Soil metal fractions and multivariate indicator profiles. (**A**,**B**) Pb and Zn fractions determined by BCR sequential extraction: F1, acid-soluble/exchangeable; F2, reducible; F3, oxidizable; F4, residual. Percentages are relative to the sum of F1–F4. (**C**,**D**) DTPA-extractable Pb and Zn; DTPA, diethylenetriaminepentaacetic acid. (**E**,**G**) Orthogonal partial least squares discriminant analysis (OPLS-DA) of nutritional and metal-related indicators, respectively; ellipses show 95% confidence regions. R^2^, model fit; Q^2^, cross-validated predictive performance. (**F**,**H**) Corresponding comprehensive scores, calculated as the mean of VIP-weighted membership scores: C score, nutritional score; HM C score, metal-indicator score; VIP, variable importance in projection. Bars show means ± SD; violins show score distributions (*n* = 6). Letters compare stages within each soil compartment and fraction in (**A**–**D**) and sampling groups in (**F**,**H**). ***, *p* < 0.001.

Pb and Zn showed distinct distribution patterns at Site H (Figure 4A,D). Pb concentrations were lower in Rs than in Bs and generally followed the order roots > leaves > culms > rhizomes in plant tissues. Zn concentrations did not differ significantly between Bs and Rs and followed the order culms > roots > leaves > rhizomes. Across stages, root Pb and Zn were relatively high at H3, while leaf Pb and culm Zn were highest at H2. All organ-specific BCF values were below 1 (Figure 4B,E). Pb was retained mainly in roots, whereas Zn showed stronger transfer to culms. Culm-to-rhizome and leaf-to-culm Pb TFs exceeded 1, but Pb TFs calculated relative to roots remained below 1. For Zn, culm-to-rhizome TFs exceeded 3 and culm-to-root TFs exceeded 1 (Figure 4C,F). BCR extraction recoveries were 88.88–97.18% for Pb and 85.52–96.49% for Zn (Tables S1 and S2). The residual F4 fraction accounted for 72–78% of Pb and 54–58% of Zn in Rs, higher than the corresponding proportions in Bs. In contrast, the exchangeable F1 fractions of both metals were lower in Rs (Figure 5A,B). DTPA-extractable Pb and Zn were also consistently lower in Rs than in Bs and reached their lowest rhizosphere values at H4 (Figure 5C,D). These results indicate lower Pb and Zn lability in rhizosphere soil.

#### 3.1.4. Integrated Plant–Soil and Metal Response Patterns

PCA and OPLS-DA were used to summarize the multivariate variation in plant–soil nutritional and Pb- and Zn-related indicators. The nutritional analysis included M and H1–H4, whereas the metal analysis included only H1–H4 because the metal analyses of samples collected during October 2024–July 2025 were restricted to Site H. Both analyses clearly separated the sampling groups (Figure 5E,G and Figure S1D,E). Site M was separated from H1–H4 in the nutritional ordinations, while the four Site H stages formed distinct clusters in both nutritional and metal profiles. Ten plant–soil nutritional indicators and 12 metal-related indicators had VIP values greater than 1 (Tables S3 and S4). Among the metal-related variables, Zn culm-to-root and leaf-to-culm TFs and culm Zn-BCF had the highest VIP values, while DTPA-extractable Pb and Zn and reducible Zn also contributed to group discrimination. The VIP-weighted nutritional-quality score was highest at M and lowest at H1; within Site H, H2 and H3 had similar scores, followed by H4 (Figure 5F). The comprehensive metal-profile score was highest at H1, intermediate at H2, and lowest at H3 and H4, which did not differ significantly from each other (Figure 5H).

### 3.2. Microbiome Structure and Stage-Associated Variation

#### 3.2.1. Alpha and Beta Diversity

To characterize microbial community diversity associated with *I. decorus*, we performed fungal ITS and bacterial 16S rRNA gene amplicon sequencing (The raw amplicon sequencing data are available in the Genome Sequence Archive of the National Genomics Data Center under accession numbers NGDC GSA: CRA041052 and CRA041053, https://ngdc.cncb.ac.cn/gsa (accessed on 17 May 2026), Q30 values ranged from 94.07% to 96.21% for bacterial libraries and from 88.82% to 98.26% for fungal libraries; Tables S5 and S6). Ecological niche was the dominant source of alpha-diversity variation: Chao1 and Shannon indices were generally higher in soil than in plant organs, and Rs had higher Chao1 richness than Bs (Figure S2). Among plant organs, roots generally showed higher bacterial diversity, whereas culms and leaves showed higher fungal diversity. Site-associated differences at shoot initiation were limited mainly to Bs, and stage-associated patterns within Site H included lower bacterial diversity in Rs at H3 and lower fungal diversity in culms and leaves at H2 and H3.

**Figure 6:**
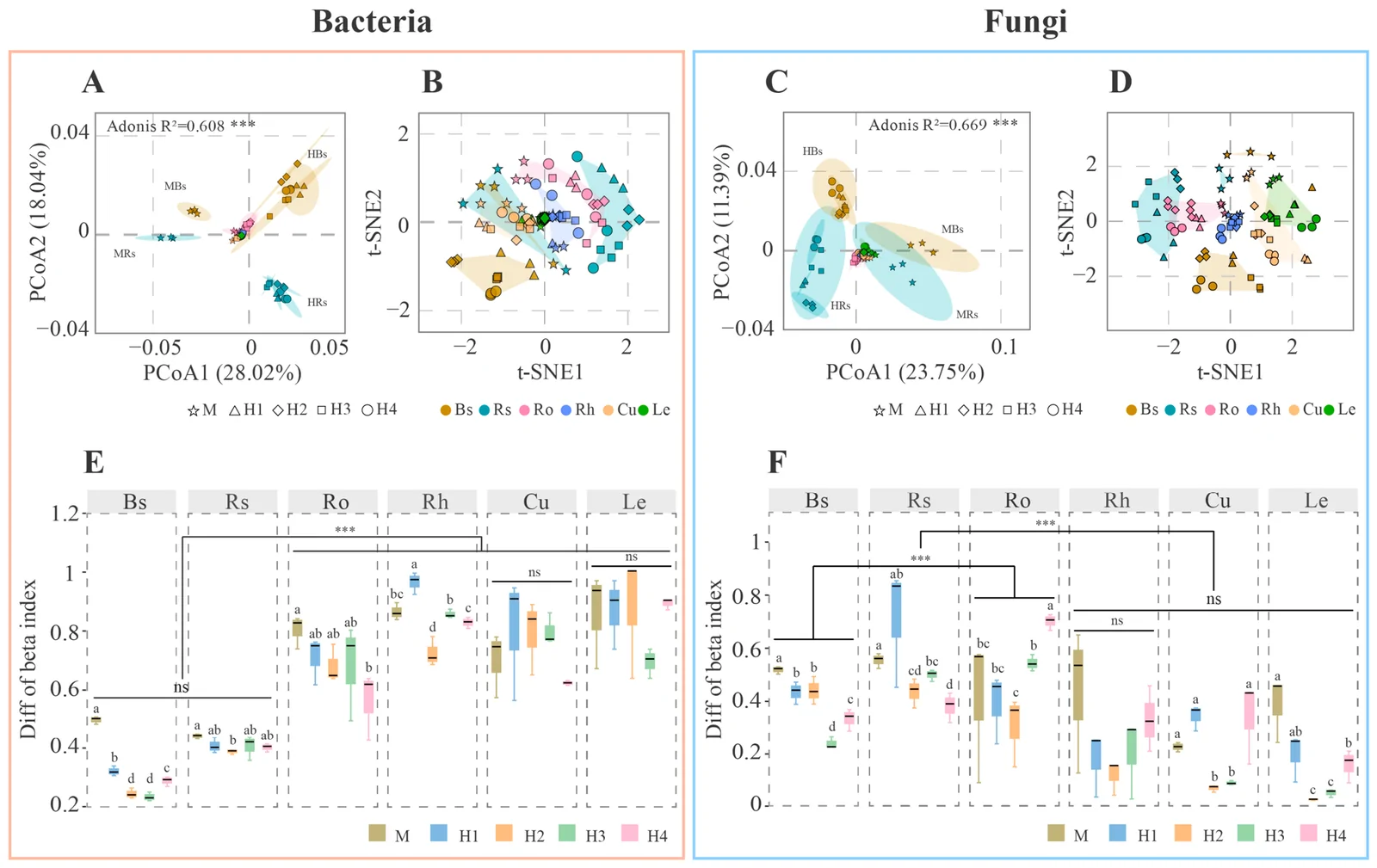
Bacterial and fungal community dissimilarities. (**A**,**C**) Principal coordinate analysis (PCoA) based on Bray–Curtis dissimilarities; ellipses show 95% confidence regions, and PERMANOVA results are displayed. PERMANOVA, permutational multivariate analysis of variance. (**B**,**D**) Corresponding t-distributed stochastic neighbour embedding (t-SNE) plots. Shapes identify sampling groups and colours identify compartments. MBs/HBs denote bulk soil from M/H; MRs/HRs denote corresponding rhizosphere samples. (**E**,**F**) Within- and between-group Bray–Curtis dissimilarities for bacteria and fungi, respectively. Three composite biological replicates were analysed per sample type and sampling group. Letters compare sampling groups within each soil compartment. ***, *p* < 0.001.

Beta-diversity analyses showed clear separation of bacterial and fungal communities among ecological niches (PERMANOVA: bacteria R^2^ = 0.608, *p* = 0.001; fungi R^2^ = 0.669, *p* = 0.001). Samples from M and Site H occupied different regions of the ordinations, while plant-organ samples formed stage-associated subclusters within Site H (Figure 6A,C). Because M was sampled at only one stage, the overall M–H separation represents a composite site-and-stage contrast. t-SNE visualization showed niche-associated clustering of bacterial and fungal communities (Figure 6B,D); because t-SNE does not preserve global distances, the two-dimensional arrangement was not interpreted as a directional ecological trajectory. Bray–Curtis dissimilarities differed between soil and plant organs for bacteria and more broadly among niches for fungi. Within Site H, stage-associated differences were detected for bacterial communities in Bs, Ro, and Rh and for fungal communities in all niches except Rh (Figure 6E,F).

#### 3.2.2. Microbial Community Composition

**Figure 7:**
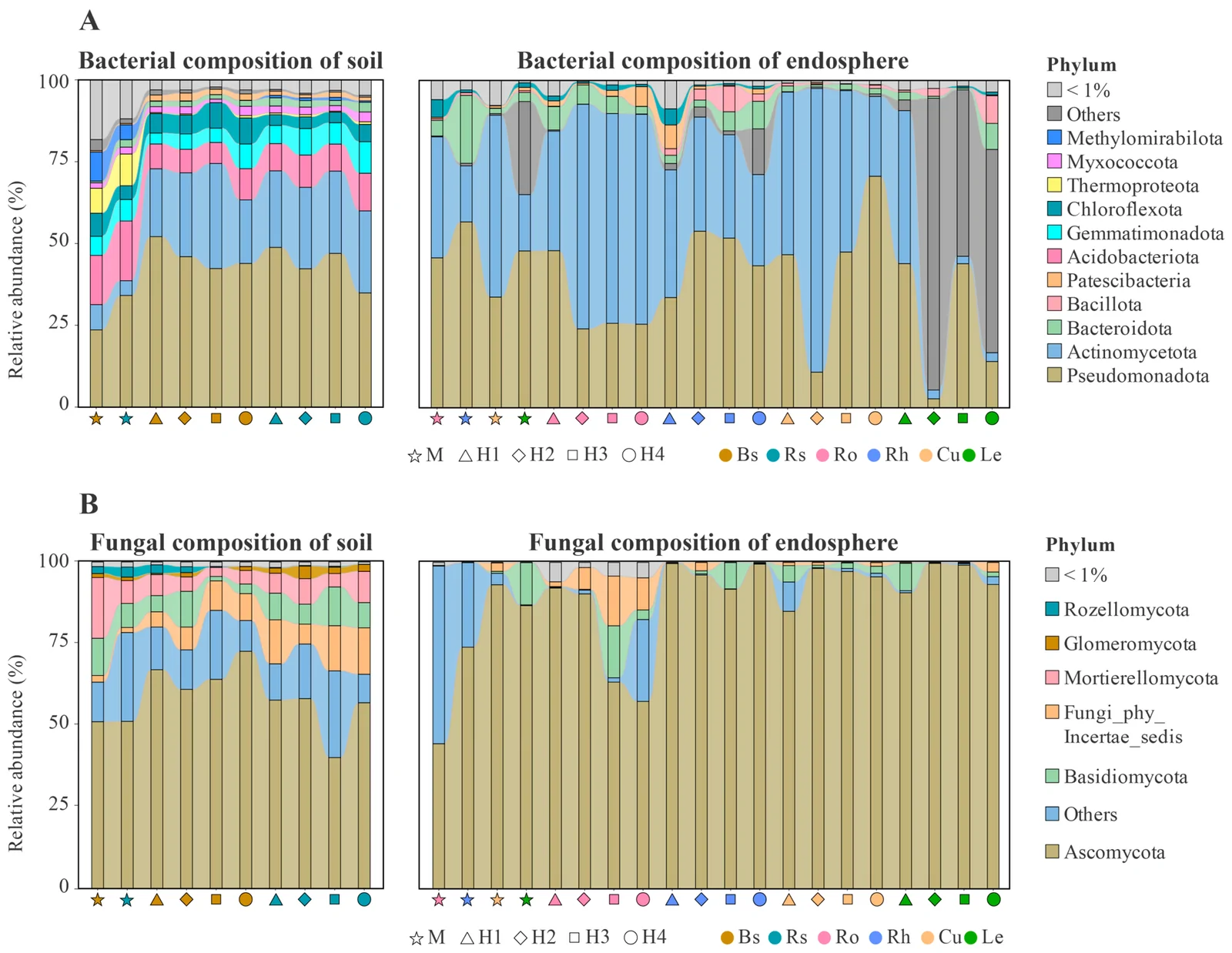
Phylum-level bacterial (**A**) and fungal (**B**) community composition. Values are mean relative abundances from three composite biological replicates per sample type and sampling group. Symbol shapes identify sampling groups and colours identify compartments.

Taxonomic analysis of rhizosphere and endosphere bacterial and fungal communities associated with *I. decorus* revealed diverse and complex community structures. At the phylum level, microbial composition differed markedly among niches, with additional variation observed within the same niche across sampling groups. For bacterial communities, soil microhabitats were dominated by Pseudomonadota, with Actinomycetota, Acidobacteriota, and Gemmatimonadota as subdominant phyla. Bacterial phyla differed markedly between M and H1: Actinomycetota had higher relative abundance at H1, whereas Acidobacteriota, Thermoproteota, and Methylomirabilota decreased. However, within the mining site, bacterial composition at the phylum level showed little variation across developmental stages. Endosphere bacterial communities in plant organs were less taxonomically diverse than soil communities, with Pseudomonadota and Actinomycetota as the core dominant phyla. Differences between M and H1 were minor, except for slight increases in Bacillota and Patescibacteria at H1. Endosphere bacterial composition also showed clear shifts across developmental stages (Figure 7A). For fungal communities, soil microhabitats were dominated by Ascomycota, with Basidiomycota, Mortierellomycota, and Fungi_phy_Incertae_sedis as subdominant phyla. Differences between M and H1 were small, with only slight increases in Fungi_phy_Incertae_sedis and Basidiomycota at H1. Endosphere fungal communities in plant organs were less taxonomically diverse than soil communities, with Ascomycota as the absolute dominant phylum. Some differences were observed between M and H1, and endosphere fungal composition varied notably across developmental stages (Figure 7B).

#### 3.2.3. Site-Associated Microbiome Differences at the Shoot-Initiation Stage

**Figure 8:**
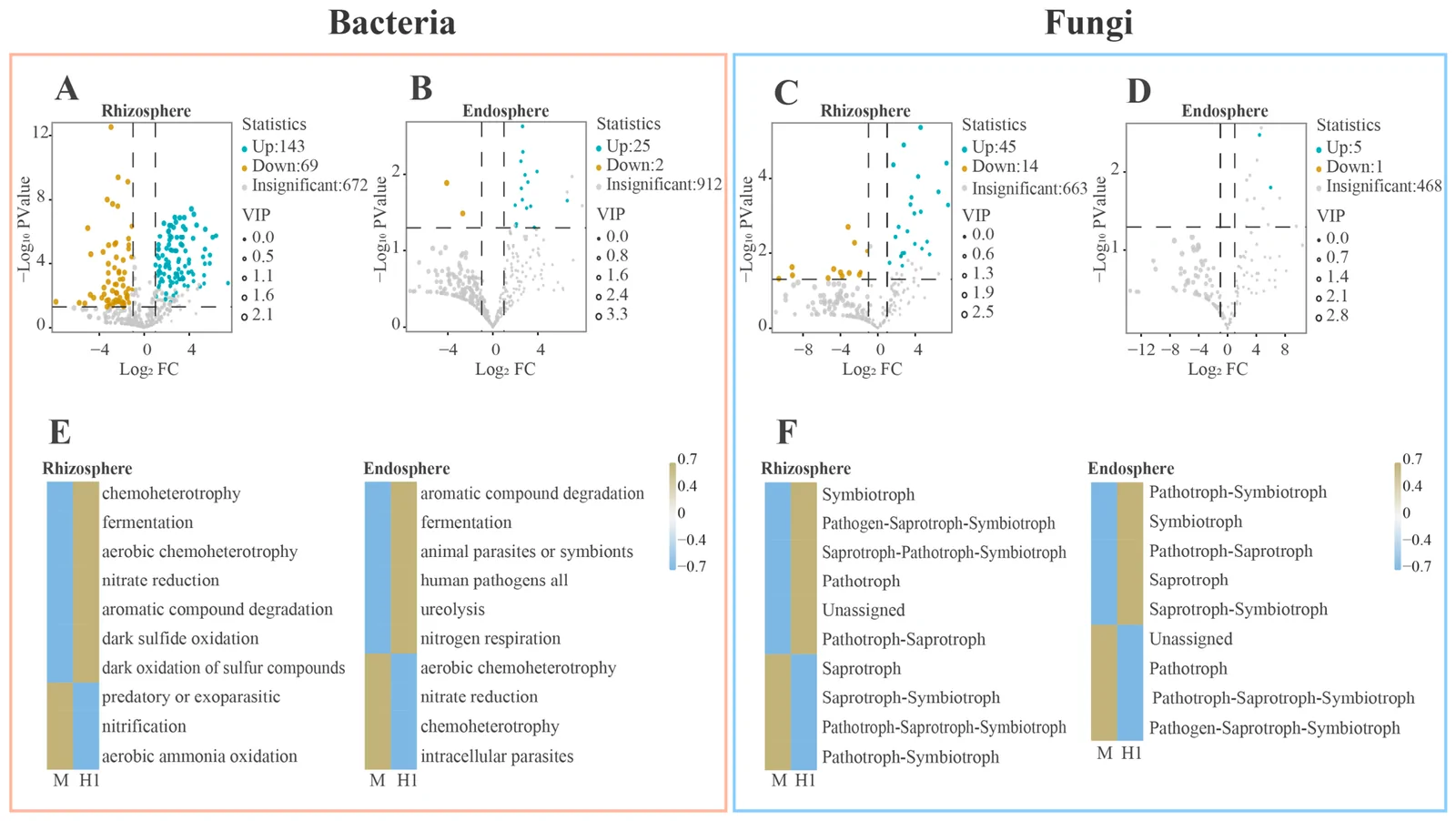
Microbial genera and predicted functions differing between M and H1. (**A**–**D**) Volcano plots for rhizosphere bacteria, endosphere bacteria, rhizosphere fungi, and endosphere fungi, respectively. Screening used Welch’s *p* < 0.05, VIP > 1, and fold change (FC; H1/M) > 2 or <0.5. Turquoise/ochre points indicate higher abundance at H1/M; grey points do not meet the criteria. Point size represents VIP; dashed lines mark *p*-value and FC thresholds. (**E**,**F**) Bacterial functions predicted by FAPROTAX and fungal trophic assignments from FUNGuild. Heatmap colours show row-wise Z-scores; warmer colours indicate higher relative abundance within each row. Analyses used three composite replicates per sample type and site. Functional assignments are taxonomic predictions, not measured activities.

At the genus level (Figure 8A–D), 67.45% of differential bacterial genera in rhizosphere soil and 92.59% in endosphere samples had higher predicted relative abundances in H1. Fifteen endosphere bacterial genera (60%), including *Streptomyces*, *Chryseobacterium*, *Pseudomonas*, and *Bacillus*, were also relatively enriched in the H1 rhizosphere. Three endosphere fungal genera (60%), including Agaricales_gen_Incertae_sedis, *Fusarium*, and *Penicillium*, showed a similar cross-compartment pattern (Tables S7–S10). The larger number of differential genera in the rhizosphere indicates greater responsiveness to the differences between M and H1, whereas enrichment at H1 identifies site-associated candidates but does not demonstrate selection by heavy metals alone.

LEfSe analysis identified four bacterial genera, including unclassified_Nitrososphaeraceae, as rhizosphere biomarkers of M, while *Sphingomonas* was a rhizosphere biomarker of H1. *Actinomycetospora* and *Sphingopyxis* were identified as endosphere bacterial biomarkers of H1 (Figure S3A). Among fungi, *Talaromyces* and *Zymoseptoria* were associated with the M rhizosphere, whereas six genera, including *Myrmecridium* and *Fusarium*, were associated with the H1 rhizosphere; no differential endosphere fungal biomarkers were detected (Figure S3B).

Predicted functional profiles also differed between the sample sets. FAPROTAX assigned higher relative abundances of chemoheterotrophy, fermentation, and nitrate reduction to the H1 rhizosphere, whereas predation or exoparasitism, nitrification, and aerobic ammonia oxidation had higher predicted relative abundances at M. Predicted endosphere bacterial functions included aromatic-compound degradation, fermentation, and nitrogen respiration at H1, and aerobic chemoheterotrophy, nitrate reduction, and intracellular parasitism at M (Figure 8E). FUNGuild assignments indicated shifts among symbiotrophic, saprotrophic, pathotrophic, and mixed trophic guilds (Figure 8F). These assignments represent predicted ecological potentials rather than measured gene expression or confirmed effects on the host, and several enriched guilds were associated with pathogenic or parasitic lifestyles.

#### 3.2.4. Microbial Community Variation Across Developmental Stages at Site H

**Figure 9:**
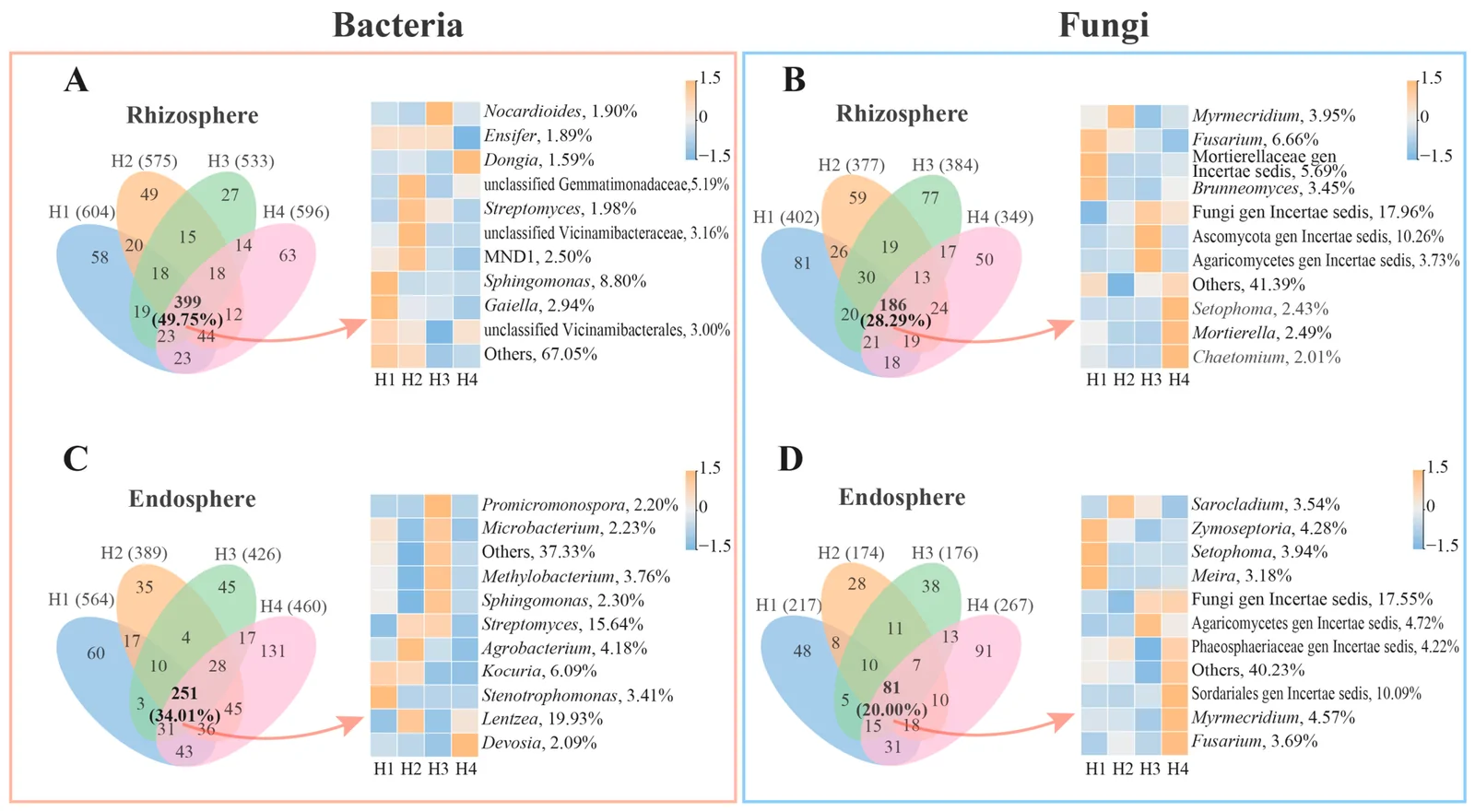
Shared and stage-specific microbial genera across H1–H4. (**A**,**B**) Rhizosphere bacteria and fungi, respectively. (**C**,**D**) Corresponding endosphere communities. Venn numbers indicate genus counts; intersection percentages are relative to the union across stages. Heatmaps show the ten most abundant shared genera, with the remainder grouped as “Others”. Label percentages indicate mean relative abundances across H1–H4.

We analyzed microbial-community patterns across the four developmental stages at Site H. Genus-level Venn diagrams showed that the proportion of shared bacterial genera was higher than that of shared fungal genera. Shared genera represented 49.75% (399) of rhizosphere bacteria and 28.29% (186) of rhizosphere fungi, while shared endosphere genera represented 34.01% (251) of bacteria and 20.00% (81) of fungi. Stage-specific genera accounted for a larger proportion of endosphere communities than of rhizosphere communities (Figure 9A–D).

Heatmaps of the ten most abundant shared genera showed clear stage-associated shifts. In the rhizosphere, *Sphingomonas* (8.80%), unclassified Vicinamibacterales (3.00%), and *Gaiella* (2.94%) were relatively abundant at H1; unclassified Gemmatimonadaceae (5.19%), unclassified Vicinamibacteraceae (3.16%), MND1 (2.50%), and *Streptomyces* (1.98%) were relatively abundant at H2. Endosphere bacteria were most strongly represented at H3 by *Streptomyces* (15.64%), *Methylobacterium* (3.76%), *Sphingomonas* (2.30%), *Microbacterium* (2.23%), and *Promicromonospora* (2.20%). Rhizosphere fungal genera showed different stage-associated peaks: *Fusarium*, *Mortierella*ceae_gen_Incertae_sedis, and *Brunneomyces* at H1; Fungi_gen_Incertae_sedis, Ascomycota_gen_Incertae_sedis, and Agaricomycetes_gen_Incertae_sedis at H3; and *Mortierella*, *Setophoma*, and *Chaetomium* at H4. Endosphere fungi at H4 included high relative abundances of Phaeosphaeriaceae_gen_Incertae_sedis, Sordariales_gen_Incertae_sedis, *Myrmecridium*, and *Fusarium* (Figure 9A–D).

Predicted functional profiles varied among compartments and stages. Across stages, 32 predicted rhizosphere bacterial functions (74.44%), 8 endosphere bacterial functions (53.33%), 30 rhizosphere fungal guilds (58.82%), and 15 endosphere fungal guilds (45.46%) were shared (Figure S3C–F). In the rhizosphere, bacterial functions such as fermentation, cellulolysis, nitrate reduction, chemoheterotrophy, and aerobic chemoheterotrophy were relatively abundant at H3, whereas aromatic compound degradation and sulfur oxidation functions were relatively abundant at H1. Endosphere bacterial predictions were characterized by chemoheterotrophy and aerobic chemoheterotrophy at H1 and by ureolysis and aromatic-compound degradation at H3. Rhizosphere fungal assignments at H2 included wood saprotrophy, arbuscular mycorrhizal fungi, and animal pathogens. Endosphere fungal assignments at H4 included undefined saprotrophs, plant pathogens, animal pathogens, plant-pathogen–wood-saprotroph guilds, and ectomycorrhizal fungi. These profiles should be treated as hypotheses for functional validation rather than direct measurements of microbial activity.

### 3.3. Plant–Soil–Metal–Microbiome Associations

**Figure 10:**
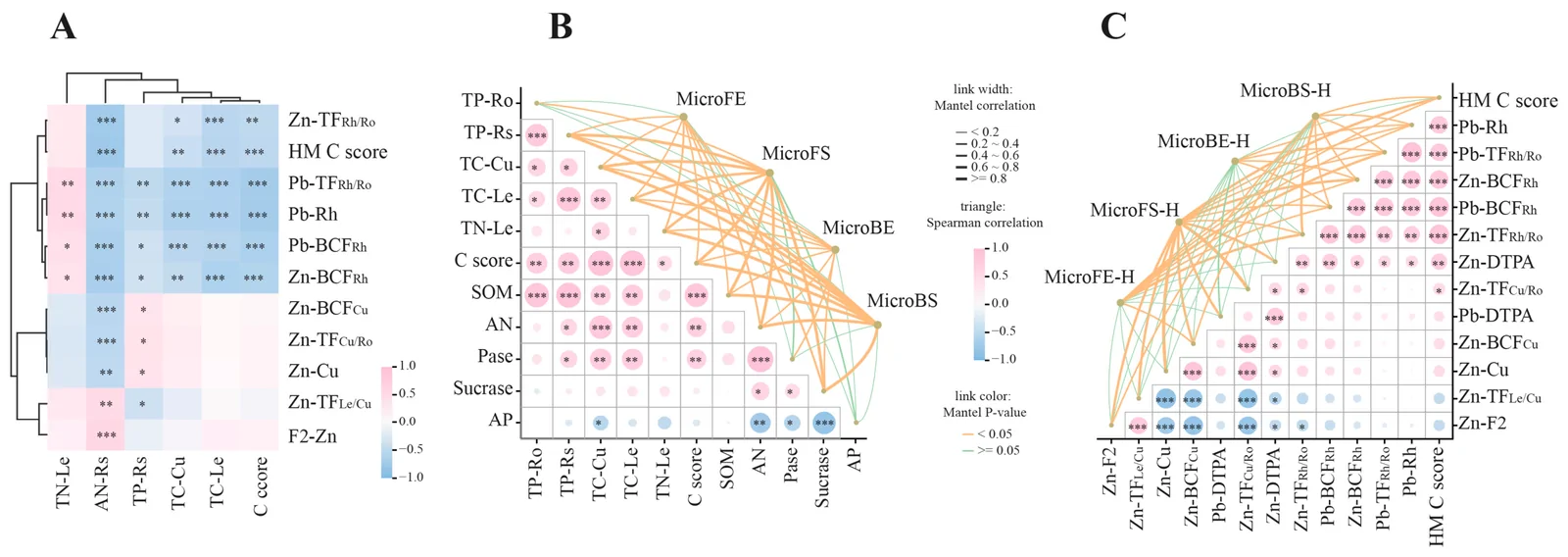
Associations among nutritional indicators, metal indicators, and microbial communities. (**A**) Spearman correlations; colours indicate correlation coefficients. (**B**,**C**) Mantel associations with nutritional and metal indicators, respectively. MicroBS/MicroFS, rhizosphere bacterial/fungal communities; MicroBE/MicroFE, root-endosphere bacterial/fungal communities; “–H”, Site H subset. *, *p* < 0.05; **, *p* < 0.01; ***, *p* < 0.001.

Spearman correlation analysis identified coordinated variation among plant nutrients, rhizosphere properties, and metal-distribution indicators (Figure 10A). Rhizosphere AN was negatively correlated with the comprehensive metal-profile score, rhizome Pb concentration, Pb translocation from roots to rhizomes, and the rhizome BCFs of Pb and Zn, but was positively correlated with reducible Zn (F2-Zn). Leaf TC and the nutritional-quality score were also negatively correlated with several Pb and Zn enrichment and translocation indices. TP in rhizosphere soil showed comparatively weaker and less consistent relationships with the metal indicators. These patterns indicate that higher rhizosphere nutrient status was generally associated with lower plant metal enrichment and internal transfer, although the direction differed among individual Zn fractions and translocation indices. Mantel tests showed that rhizosphere bacterial and fungal communities and root-endosphere bacterial and fungal communities were significantly associated with multiple plant and soil indicators (Mantel *p* < 0.05; Figure 10B).

Within Site H, microbial communities were also associated with Pb and Zn fractionation, extractability, and plant-distribution indices (Figure 10C). Significant Mantel associations involved DTPA-extractable Pb and Zn, reducible Zn, rhizome Pb concentration, organ-specific BCFs, and within-plant TFs. Rhizosphere fungi were associated with the broadest set of metal indicators, whereas root-endosphere communities showed more selective relationships with Zn fractionation and rhizome metal enrichment and transfer. The metal indicators themselves formed contrasting correlation groups: the comprehensive metal-profile score covaried positively with rhizome Pb concentration, Pb translocation to rhizomes, and several rhizome BCFs, while reducible Zn and several culm-related Zn indices showed opposing relationships.

## 4. Discussion

### 4.1. Plant Nutrient Patterns and Rhizosphere Stabilization of Pb and Zn Under Mine-Site Conditions

Plant establishment in metal-contaminated soils requires coordinated nutrient acquisition, biomass production, and metal tolerance. Excess metals can disrupt nutrient uptake and allocation [29]. At shoot initiation, *I. decorus* at Site H had lower biomass, generally lower P, and lower culm and leaf N than plants at Site M. Root N concentration did not differ significantly between M and H1. This may sustain root activity and resource acquisition during early establishment [30]. Rhizome and culm C remained high during shooting and branch-and-leaf expansion. These concentration patterns may be related to the high C demand of bamboo shoot and structural growth [31]. Root and rhizome N and P also varied across stages, but nutrient allocation among organs was not quantified [8]. The rhizosphere likely supported this nutrient adjustment. As the root–soil–microbe interface, it often differs from bulk soil [32,33]. Here, rhizosphere soil generally had more available nutrients and higher enzyme activities than bulk soil. At Site H, SOM, TN, TP, and AP were relatively high at H2, whereas AN was highest at H3. Enzyme responses were not uniform: H3 had high phosphatase activity, relatively high urease, sucrase, and β-glucosidase activities, but low FDAase activity. This suggests that C-, N-, and P-related processes responded differently, rather than showing a uniform change in microbial activity. Similar nutrient–microbe–enzyme covariation has been reported [31]. These M–H1 contrasts are site-associated: elevation, temperature, rainfall, soil fertility, and contamination differed simultaneously between the two sites.

Pb and Zn distributions and fractions also differed between bulk and rhizosphere soils. Compared with bulk soil, rhizosphere soil had lower total Pb and lower DTPA-extractable Pb and Zn, a smaller proportion of F1, and a larger proportion of residual F4. These operational measurements are consistent with lower extractability near roots, but do not identify the processes responsible. Plant uptake, root-associated microbes, and rhizosphere chemistry may promote metal adsorption or precipitation [34,35,36]. Microbial Pb immobilization can involve cell-surface sorption and phosphate-, sulfide-, or carbonate-containing precipitates. Pb and Zn also showed element-specific distribution in plants. Pb accumulated mainly in roots and remained low in rhizomes, whereas Zn was highest in culms and moved upward more readily. This Pb pattern differs from the “old-culm dominance and rhizome synergy” reported for *Indocalamus* under single-Pb exposure, possibly due to contamination mixtures, soil conditions, and plant establishment [37]. Still, BCF values below 1 for both metals, Pb retention in roots, and the shift toward less labile rhizosphere fractions indicate limited tissue enrichment and a phytostabilization-oriented response [34]. Together, root-dominated Pb retention, limited tissue enrichment, and lower rhizosphere metal lability support further evaluation of *I. decorus* for phytostabilization-oriented management [5,38].

### 4.2. Rhizosphere–Endosphere Differentiation and Microbiome Variation Among Sampling Stages

Sample compartment accounted for a large proportion of the variation in bacterial and fungal community composition associated with *I. decorus*, explaining 60.8% and 66.9% of Bray–Curtis variation, respectively. Soil samples had greater microbial richness than plant tissues. Roots had relatively diverse bacterial communities; culms and leaves had greater fungal diversity. This pattern fits sequential filtering from bulk soil to the rhizosphere and then into plant tissues [39]. Soil history and physicochemical properties shape the available microbial pool. Root-derived substrates modify the rhizosphere community. Entry into the endosphere imposes additional host-associated selection [40,41,42]. Thus, rhizosphere communities remained closely associated with the surrounding soil, while endosphere communities formed distinct, tissue-dependent assemblages [33,43]. At shoot initiation, microbial differences between M and H1 were stronger in the rhizosphere than in the endosphere, indicating that the root–soil interface was especially responsive to contrasting site conditions [44,45]. Higher relative abundance of Actinomycetota and many bacterial and fungal genera at H1 suggests a distinct microbial assemblage under mining-site nutrient and metal conditions. Several H1-associated taxa, including *Sphingomonas*, *Actinomycetospora*, and *Sphingopyxis*, have been reported in stressful or metal-affected environments, though their ecological functions vary by species and strain [41,46,47]. Predicted functional profiles also differed. Taxa assigned by FAPROTAX to nitrification and aerobic ammonia oxidation had higher relative abundances in the M rhizosphere, whereas those assigned to chemoheterotrophy, fermentation, and nitrate reduction had higher predicted relative abundances at H1. These taxonomic predictions suggest differences in potential C and N transformation functions between the sites, rather than measured changes in microbial activity. Shifts among fungal symbiotrophic, saprotrophic, and pathotrophic guilds further indicate that site conditions may alter multiple ecological components of the plant-associated fungal community [48,49].

Microbial communities at Site H also changed across the four sampling stages. Bacterial communities shared more genera than fungal communities. Stage-specific genera were more common in the endosphere than in the rhizosphere. This suggests a relatively stable bacterial core, while fungal and endosphere communities showed greater variation across sampling stages. At H2, enrichment of rhizosphere taxa such as unclassified Gemmatimonadaceae, unclassified Vicinamibacteraceae, MND1, and *Streptomyces* coincided with relatively high rhizosphere nutrient concentrations. H3 had higher relative abundance of several endosphere bacterial genera, including *Streptomyces*, *Methylobacterium*, *Sphingomonas*, *Microbacterium*, and *Promicromonospora*, along with high phosphatase activity. Predicted rhizosphere bacterial functions related to aromatic-compound degradation and sulfur oxidation were relatively abundant at H1, whereas heterotrophy, cellulolysis, fermentation, and nitrate reduction were relatively abundant at H3. At H4, endosphere fungal communities showed greater representation of saprotrophic assignments. These coincident patterns may reflect changes in plant nutrient demand, tissue properties, and rhizosphere resource inputs [50,51,52], but developmental and seasonal contributions cannot be separated in this design. The endosphere-associated taxa identified here also provide a resource for culture-based screening. H1-associated *Actinomycetospora* and *Sphingopyxis*, plus genera found in both the rhizosphere and endosphere such as Pseudomonas, Bacillus, and Streptomyces, include strains previously reported to have metal tolerance, nutrient mobilization, or plant-growth-promoting traits [53,54,55]. In contrast, candidates assigned to *Fusarium* need careful taxonomic and functional evaluation because this genus includes beneficial, neutral, and pathogenic species [56]. Isolation, strain-level characterization, and reinoculation experiments could determine whether these resident microorganisms contribute to nutrient acquisition or metal tolerance in *I. decorus*. Compatible native strains may also help construct synthetic communities, an approach that has improved plant performance under mixed metal (loid) stress in other systems [57].

### 4.3. Associations Among Plant Nutrition, Rhizosphere Properties, Metal Distribution, and Microbial Communities

Coordinated variation in plant nutrients, rhizosphere properties, metal partitioning, and microbial communities indicates that *I. decorus* responses at the mining site involved linked plant–soil processes. Rhizosphere available N was negatively correlated with the comprehensive metal-profile score and several Pb and Zn enrichment or translocation indices, but positively correlated with the Zn F2 fraction. Leaf C and the comprehensive nutritional-quality score were also negatively associated with several metal enrichment and transfer indices. These relationships suggest that plant nutrient status and rhizosphere nutrient availability were closely linked to Pb and Zn distribution between soil fractions and plant organs. The patterns do not reflect a simple increase or decrease in metal uptake; they describe covariation among nutrient concentrations, belowground metal concentrations, and internal concentration ratios. Microbial–environment associations were strongly compartment dependent. Rhizosphere fungal communities were associated with a broader range of metal indicators, including DTPA-extractable Pb and Zn, Zn fractions, and plant BCF and TF indices. Bacterial and endosphere communities showed more selective associations. This difference reflects the distinct ecological positions of rhizosphere and endosphere microorganisms [33]. Rhizosphere fungi directly contact roots and soil minerals and may affect metal availability through hyphal sorption, extracellular polymers, organic-acid production, mineral weathering, and pH changes. Endosphere microorganisms are more directly linked to host nutrient metabolism, stress responses, and metal transport or sequestration within plant tissues. Related studies show that root-associated microorganisms can alter metal mobility, regulate host metal transporters, enhance antioxidant responses, and influence aboveground metal accumulation [58,59].

Developmental-stage patterns further support this plant–soil–microbiome linkage. H2 combined relatively high rhizosphere nutrient availability with shifts in rhizosphere taxa during shooting. At H3, high phosphatase activity coincided with enrichment of several endosphere bacterial genera during branch-and-leaf expansion. At H4, higher biomass than at H1, relatively high β-glucosidase activity, and greater representation of predicted endosphere saprotrophic guilds suggested hypotheses about C turnover during rhizome extension, which was not directly measured. Thus, the four stages differed in their combinations of plant nutrient concentrations, rhizosphere biochemical activity, metal distribution, and microbial composition, rather than following one uniform response pathway. Together, these findings suggest a compartment-specific association pattern: rhizosphere microorganisms, especially fungi, are closely associated with root-zone metal availability and fractionation, while endosphere microorganisms are more closely linked to metal distribution and transfer within the plant. Root microbiota can influence plant development and stress responses in other systems [60], providing a basis for testing the candidate taxa identified here. Native core microorganisms may establish and promote plant growth more consistently than non-native or transient taxa because they are adapted to the host and local soil [61]. Therefore, isolating and functionally screening H1- and stage-associated taxa, followed by reinoculation and compatibility tests, could identify strains or simplified consortia that support nutrient acquisition, Pb retention, or Zn regulation in *I. decorus* under mine-site conditions [62].

### 4.4. Limitations

This study has several limitations. First, the site comparison was restricted to M versus H1 at shoot initiation. The three composite replicates represent within-site biological sampling rather than replication of provenance and mining sites. Because only one site of each type was studied, and the sites differed in elevation, temperature, rainfall, and baseline soil fertility, site-associated differences cannot be attributed exclusively to Pb–Zn contamination. Second, clean exogenous soil was mixed into the contaminated surface layer before planting, but the initial post-mixing metal stock was not quantified; temporal metal removal and remediation efficiency therefore cannot be estimated. Third, although biomass was measured at M, H1, and H4, biomass-weighted metal stocks were not quantified across all stages, and whole-system metal mass balances could not be calculated. Fourth, FAPROTAX and FUNGuild assignments represent predicted ecological potentials rather than gene expression or measured activity [49]. Fifth, correlations do not establish causality, and candidate taxa require isolation, reinoculation, and functional validation [63]. Finally, developmental stages were sampled sequentially in different months, so stage-associated patterns also include potential seasonal variation. Because clumps were randomly resampled from the same established stand and pooled independently at each stage rather than tracked as fixed longitudinal units, the results describe stand-level patterns rather than within-clump trajectories. Repeated multi-year sampling with environmental covariates is needed to evaluate temporal stability and disentangle developmental from seasonal effects [64].

## 5. Conclusions

*Indocalamus decorus* showed stage-associated changes in nutrient concentrations and rhizosphere properties under Pb–Zn mine-site conditions, with relatively high rhizosphere nutrient availability during shooting. Pb was retained predominantly in roots, whereas Zn showed greater transfer to culms, and organ-specific BCF values for both metals remained below 1. Compared with bulk soil, rhizosphere soil contained lower DTPA-extractable Pb and Zn, smaller proportions of the exchangeable F1 fraction, and larger proportions of the residual F4 fraction. These patterns indicate limited plant enrichment and lower rhizosphere metal lability, supporting further evaluation of *I. decorus* as a candidate for phytostabilization-oriented management rather than demonstrating effective phytoextraction or long-term stabilization.

Bacterial and fungal community composition differed strongly among sample compartments, while stage-associated changes occurred in both the rhizosphere and plant endosphere. Rhizosphere fungi were associated with a broader range of soil metal fractions and plant metal-distribution indices, whereas endosphere communities showed more selective associations with internal metal distribution and transfer. These results show compartment-specific plant–soil–microbiome covariation and identify native microbial taxa for subsequent isolation and functional validation.

## Figures and Tables

**Table 1:** Soil physicochemical properties and Pb and Zn concentrations.

Soil	Pb (mg kg^−1^)	Zn (mg kg^−1^)	pH	SOM (g kg^−1^)	AN (mg kg^−1^)	AP (mg kg^−1^)
Tailing soil	1285.42 ± 73.16	1045.83 ± 52.08	7.34 ± 0.26	7.54 ± 0.43	8.75 ± 1.12	3.04 ± 0.26
Provenance site soil	41.26 ± 2.57	32.81 ± 1.14	7.63 ± 0.47	23.76 ± 3.68	44.82 ± 5.16	18.75 ± 0.16
Clean exogenous soil	103.27 ± 8.34	114.09 ± 8.82	7.71 ± 0.31	20.71 ± 2.74	42.08 ± 3.45	20.63 ± 0.61

Note: Values are means ± standard deviation (SD; *n* = 6). SOM, soil organic matter; AN, available nitrogen; AP, available phosphorus. Concentrations are expressed on a dry-soil basis.

## Data Availability

The raw bacterial and fungal amplicon sequencing data generated in this study are publicly available in the Genome Sequence Archive of the National Genomics Data Center under accession numbers CRA041052 and CRA041053 (https://ngdc.cncb.ac.cn/gsa, accessed on 17 May 2026). Other data supporting the findings of this study are included in the article and its Supplementary Materials. Further inquiries can be directed to the corresponding author.

## Supplementary Materials

The following supporting information can be downloaded at: https://www.mdpi.com/article/10.3390/toxics14100897/s1, Figure S1: Additional soil measurements, multivariate profiles, and biomass; Figure S2: Microbial alpha diversity across compartments and sampling groups; Figure S3: Microbial biomarkers and predicted functional profiles; Table S1: Pb concentrations in BCR fractions and extraction recoveries; Table S2: Zn concentrations in BCR fractions and extraction recoveries; Table S3: VIP-weighted membership scores of nutritional indicators; Table S4: VIP-weighted membership scores of metal indicators; Table S5: Bacterial amplicon sequencing statistics; Table S6: Fungal amplicon sequencing statistics; Table S7: Top 20 rhizosphere bacterial genera enriched at H1 relative to M, ranked by VIP; Table S8: Top 20 rhizosphere fungal genera enriched at H1 relative to M, ranked by VIP; Table S9: Endosphere bacterial genera enriched at H1 relative to M, ranked by VIP; Table S10: Endosphere fungal genera enriched at H1 relative to M, ranked by VIP.

## References

[1] Hou D., Jia X., Wang L., McGrath S.P., Zhu Y.-G., Hu Q., Zhao F.-J., Bank M.S., O’Connor D., Nriagu J. (2025). Global Soil Pollution by Toxic Metals Threatens Agriculture and Human Health. Science.

[2] Zhao B., Sun S., Zhao W., Mao H., Tang Q., Zhu S. (2025). Ecological Risk assessment of Soil Heavy Metals in Typical Lead-zinc Mining Areas and Rhizosphere Microbial Response of Miscanthus. Environ. Chem..

[3] Rani M., Vikas, Kumar R., Lathwal M., Kamboj A., Kumar N., Jha A.K. (2024). Effect and Responses of Lead Toxicity in Plants. Lead Toxicity Mitigation: Sustainable Nexus Approaches.

[4] Dhiman S.S., Selvaraj C., Li J., Singh R., Zhao X., Kim D., Kim J.Y., Kang Y.C., Lee J.-K. (2016). Phytoremediation of Metal-Contaminated Soils by the Hyperaccumulator Canola (*Brassica napus* L.) and the Use of Its Biomass for Ethanol Production. Fuel.

[5] Bian F., Zhong Z., Zhang X., Yang C., Gai X. (2020). Bamboo—An Untapped Plant Resource for the Phytoremediation of Heavy Metal Contaminated Soils. Chemosphere.

[6] Lima R.A.F., Rother D.C., Muler A.E., Lepsch I.F., Rodrigues R.R. (2012). Bamboo Overabundance Alters Forest Structure and Dynamics in the Atlantic Forest Hotspot. Biol. Conserv..

[7] Kumar V., Singh K., Shah M.P., Kumar M., Kumar V., Saxena G., Shah M.P. (2021). Chapter 22—Phytocapping: An Eco-Sustainable Green Technology for Environmental Pollution Control. Bioremediation for Environmental Sustainability.

[8] Miao Z., Tang D., Jiang M., Chen G., Wang K., Zheng Y., Lyu B., Luo Z. (2026). Comprehensive Analysis of the Adaptation Mechanism among Growth Strategies, Rhizosphere Effects, and Microbial Community of Three *Indocalamus* Species. Ind. Crops Prod..

[9] Yang Y., Jiang M., Liao J., Luo Z., Gao Y., Yu W., He R., Feng S. (2022). Effects of Simultaneous Application of Double Chelating Agents to Pb-Contaminated Soil on the Phytoremediation Efficiency of *Indocalamus decorus* Q. H. Dai and the Soil Environment. Toxics.

[10] Yang Y., Jiang M., Chen C., Wang S., Song G., Gao Y., Robinson B.H., Luo Z. (2025). The Phytomanagement of Pb-Contaminated Soil by *Indocalamus decorus* through Mowing and Inoculation of *Acidithiobacillus thiooxidans*. J. Environ. Chem. Eng..

[11] Sachdev S., Keswani C., Minkina T., Bauddh K. (2025). Mechanisms of Microbe-Assisted Metal Tolerance in Phytoremediators: A Review. Pedosphere.

[12] Li Q., Yao S., Wen H., Li W., Jin L., Huang X. (2024). Improving Lead Phytoremediation Using Endophytic Bacteria Isolated from the Pioneer Plant *Ageratina adenophora* (Spreng.) from a Mining Area. Toxics.

[13] Tovar-Sanchez E., Concepcion-Acosta C.M., Sanchez-Reyes A., Sanchez-Cruz R., Folch-Mallol J.L., Mussali-Galante P. (2023). *Aspergillus luchuensis*, an Endophyte Fungus from the Metal Hyperaccumulator Plant *Prosopis laevigata*, Promotes Its Growth and Increases Metal Translocation. Plants.

[14] George S.E., Wan Y. (2023). Microbial Functionalities and Immobilization of Environmental Lead: Biogeochemical and Molecular Mechanisms and Implications for Bioremediation. J. Hazard. Mater..

[15] Klesta E.J., Bartz J.K. (1996). Part 3: Chemical Methods. Methods of Soil Analysis.

[16] Ge J., Xie Z. (2017). Leaf Litter Carbon, Nitrogen, and Phosphorus Stoichiometric Patterns as Related to Climatic Factors and Leaf Habits across Chinese Broad-Leaved Tree Species. Plant Ecol..

[17] Dick R.P. (2011). Methods of Soil Enzymology.

[18] Lin S., Wang S., Si Y., Yang W., Zhu S., Ni W. (2017). Variations in Eco-Enzymatic Stoichiometric and Microbial Characteristics in Paddy Soil as Affected by Long-Term Integrated Organic-Inorganic Fertilization. PLoS ONE.

[19] Liu C., Song Y., Dong X., Wang X., Ma X., Zhao G., Zang S. (2021). Soil Enzyme Activities and Their Relationships with Soil C, N, and P in Peatlands from Different Types of Permafrost Regions, Northeast China. Front. Environ. Sci..

[20] Green V.S., Stott D.E., Diack M. (2006). Assay for Fluorescein Diacetate Hydrolytic Activity: Optimization for Soil Samples. Soil. Biol. Biochem..

[21] Tabatabai M.A., Bremner J.M. (1969). Use of p-Nitrophenyl Phosphate for Assay of Soil Phosphatase Activity. Soil. Biol. Biochem..

[22] Gao Y., Jiang M., Tian Y., Yang Y., Luo Z., Lyu B., Chen Q. (2023). Risk-Spreading Strategies of Two Dwarf Bamboos in Heterogeneous Pb (Lead) Environments. Glob. Ecol. Conserv..

[23] Rauret G., López-Sánchez J.F., Sahuquillo A., Rubio R., Davidson C., Ure A., Quevauviller P. (1999). Improvement of the BCR Three Step Sequential Extraction Procedure Prior to the Certification of New Sediment and Soil Reference Materials. J. Environ. Monit..

[24] Xiao L., Li M., Dai J., Motelica-Heino M., Chen X., Wu J.-L., Zhao L., Liu K., Zhang C. (2020). Assessment of Earthworm Activity on Cu, Cd, Pb and Zn Bioavailability in Contaminated Soils Using Biota to Soil Accumulation Factor and DTPA Extraction. Ecotoxicol. Environ. Saf..

[25] Van Der Maaten L. (2014). Accelerating T-SNE Using Tree-Based Algorithms. J. Mach. Learn. Res..

[26] R Core Team (2023). R: A Language and Environment for Statistical Computing.

[27] Thévenot E.A., Roux A., Xu Y., Ezan E., Junot C. (2015). Analysis of the Human Adult Urinary Metabolome Variations with Age, Body Mass Index, and Gender by Implementing a Comprehensive Workflow for Univariate and OPLS Statistical Analyses. J. Proteome Res..

[28] Li W., Li Y., Lv J., He X., Wang J., Teng D., Jiang L., Wang H., Lv G. (2022). Rhizosphere Effect Alters the Soil Microbiome Composition and C, N Transformation in an Arid Ecosystem. Appl. Soil. Ecol..

[29] Panda A., Fatnani D., Parida A.K. (2025). Uptake, Impact, Adaptive Mechanisms, and Phytoremediation of Heavy Metals by Plants: Role of Transporters in Heavy Metal Sequestration. Plant Physiol. Biochem..

[30] Wang R., Dijkstra F.A., Han X., Jiang Y. (2024). Root Nitrogen Reallocation: What Makes It Matter?. Trends Plant Sci..

[31] Ren P., Li P., Zhou X., Liu Z., Tang J., Zhang C., Zou Z., Li T., Peng C. (2024). Shifts in Plant Phenology Significantly Affect the Carbon Allocation in Different Plant Organs. Ecol. Lett..

[32] Garbeva P., van Elsas J.D., van Veen J.A. (2007). Rhizosphere Microbial Community and Its Response to Plant Species and Soil History. Plant Soil.

[33] Trivedi P., Leach J.E., Tringe S.G., Sa T., Singh B.K. (2020). Plant–Microbiome Interactions: From Community Assembly to Plant Health. Nat. Rev. Microbiol..

[34] Egendorf S.P., Groffman P., Moore G., Cheng Z. (2020). The Limits of Lead (Pb) Phytoextraction and Possibilities of Phytostabilization in Contaminated Soil: A Critical Review. Int. J. Phytoremediation.

[35] Liao J., Jiang M., Lu Y., Yang Y., Gao Y., Chen Q., Luo Z., Yu X. (2024). Lead Tolerance and Remediation Potential of Four *Indocalamus* Species in Lead-Contaminated Soil. Plants.

[36] Lin S., Wu B., Li X., Yang X., Lin Q., Qiu R. (2025). Pioneer Plants Enhance Heavy Metal Immobilization in Mining Soils: Decoding Rhizosphere-Driven Mechanisms. Plant Soil.

[37] Cai X., Jiang M., Liao J., Yang Y., Li N., Cheng Q., Li X., Song H., Luo Z., Liu S. (2021). Biomass Allocation Strategies and Pb-Enrichment Characteristics of Six Dwarf Bamboos under Soil Pb Stress. Ecotoxicol. Environ. Saf..

[38] Gao Y., Jiang M., Yang Y., Wang S., Chen C., Luo Z., Lyu B., Chen Q. (2025). Tolerance Mechanisms of Two Species of *Indocalamus* under Soil Pb Stress and the Effects of Rhizosphere Environmental Factors on Tolerance. Ecotoxicol. Environ. Saf..

[39] Ma S., Qiao L., Liu X., Zhang S., Zhang L., Qiu Z., Yu C. (2022). Microbial Community Succession in Soils under Long-Term Heavy Metal Stress from Community Diversity-Structure to KEGG Function Pathways. Environ. Res..

[40] Dastogeer K.M.G., Tumpa F.H., Sultana A., Akter M.A., Chakraborty A. (2020). Plant Microbiome–an Account of the Factors That Shape Community Composition and Diversity. Curr. Plant Biol..

[41] Peng M., Wang C., Wang Z., Huang X., Zhou F., Yan S., Liu X. (2022). Differences between the Effects of Plant Species and Compartments on Microbiome Composition in Two Halophyte *Suaeda* Species. Bioengineered.

[42] Samuel S.O., Suzuki K., Asiloglu R., Harada N. (2023). Rice Endophytic Communities Are Strongly Dependent on Microbial Communities Specific to Each Soil. Biol. Fertil. Soils.

[43] Ali D., Asif A., Koner S., Chang E.-H., Hussain A., Hsu B.-M. (2026). Plant-Type Shape Arbuscular Mycorrhizal Fungal Community Assembly Formation in Rhizospheric Microbiome of Long-Term Heavy Metal-Stressed Ultramafic Soil. Appl. Soil. Ecol..

[44] Xu Q., Jiang D., Zhou N., Kang Y., Li M., Yang C., Liu X., Mi J., Hua G., Ren G. (2024). Community Structure of Soil Microorganisms and Endophytes of Honeysuckle at Different Ecological Niche Specificities. BMC Microbiol..

[45] Zuo Y., Li X., Yang J., Liu J., Zhao L., He X. (2021). Fungal Endophytic Community and Diversity Associated with Desert Shrubs Driven by Plant Identity and Organ Differentiation in Extremely Arid Desert Ecosystem. J. Fungi.

[46] Li H.-Y., Li D.-W., He C.-M., Zhou Z.-P., Mei T., Xu H.-M. (2012). Diversity and Heavy Metal Tolerance of Endophytic Fungi from Six Dominant Plant Species in a Pb-Zn Mine Wasteland in China. Fungal Ecol..

[47] Strobel G. (2018). The Emergence of Endophytic Microbes and Their Biological Promise. J. Fungi.

[48] Deng Y., Xiao W., Xiong Z., Sha A., Luo Y., Chen X., Li Q. (2024). Assembly Mechanism of Rhizosphere Fungi in Plant Restoration in Lead Zinc Mining Areas. Genes..

[49] Zamkovaya T., Foster J.S., de Crécy-Lagard V., Conesa A. (2020). A Network Approach to Elucidate and Prioritize Microbial Dark Matter in Microbial Communities. ISME J..

[50] Akhtar O., Pandey D., Zoomi I., Singh U., Chaudhary K.L., Mishra R., Pandey N. (2024). Role of Arbuscular Mycorrhizal Fungi in Heavy Metals Homoeostasis in Plants. J. Plant Growth Regul..

[51] He W., Megharaj M., Wu C., Subashchandrabose S.R., Dai C. (2019). Endophyte-Assisted Phytoremediation: Mechanisms and Current Application Strategies for Soil Mixed Pollutants. Crit. Rev. Biotechnol..

[52] Zhang X., Liu Y., Cao Q., Yang Z., Yu Z., Li C., Zhang C., Dong Q. (2025). The Interaction among Rhizosphere Soil Nutrients, Metabolites, and Microbes Determines the Productivity of Perennial Cultivated Grassland in Qinghai-Tibet Plateau. Ecol. Evol..

[53] Ozkoc H.B., Aliustaoglu M.T., Senturk I. (2023). Bioremediation of Copper with Endophytic Bacteria *Bacillus* sp. and *Streptomyces griseus*. J. Environ. Eng..

[54] Sharma M., Khurana H., Singh D.N., Negi R.K. (2021). The Genus *Sphingopyxis*: Systematics, Ecology, and Bioremediation Potential—A Review. J. Environ. Manag..

[55] Singh V.K., Shukla A.K., Singh A.K. (2024). Endophytic Bacillus Species as Multifaceted Toolbox for Agriculture, Environment, and Medicine. Environ. Dev. Sustain..

[56] Sanchez C. (2024). *Fusarium* as a Promising Fungal Genus with Potential Application Bioremediation for Pollutants Mitigation: A Review. Biotechnol. Adv..

[57] Flores-Duarte N.J., Pajuelo E., Mateos-Naranjo E., Navarro-Torre S., Rodriguez-Llorente I.D., Redondo-Gomez S., Lopez J.A.C. (2023). A Culturomics-Based Bacterial Synthetic Community for Improving Resilience towards Arsenic and Heavy Metals in the Nutraceutical Plant Mesembryanthemum Crystallinum. Int. J. Mol. Sci..

[58] Aziz L., Hamayun M., Rauf M., Iqbal A., Husssin A., Khan S.A., Irshad M., Lee I. (2021). *Aspergillus flavus* Reprogrammed Morphological and Chemical Attributes of *Solanum lycopersicum* through SlGSH1 and SlPCS1 Genes Modulation under Heavy Metal Stress. J. Plant Interact..

[59] Tong J., Ye B., Jiang X., Wu H., Xu Q., Luo Y., Pang J., Jia F., Shi J. (2023). Synergy among Extracellular Adsorption, Bio-Precipitation and Transmembrane Transport of *Penicillium oxalicum* SL2 Enhanced Pb Stabilization. J. Hazard. Mater..

[60] Zhang J., Wang B., Xu H., Liu W., Yu J., Wang Q., Yu H., Wei J.-W., Dai R., Zhou J. (2025). Root Microbiota Regulates Tiller Number in Rice. Cell.

[61] Zhou Y., Liu D., Li F., Dong Y., Jin Z., Liao Y., Li X., Peng S., Delgado-Baquerizo M., Li X. (2024). Superiority of Native Soil Core Microbiomes in Supporting Plant Growth. Nat. Commun..

[62] Li X., Zheng X., Yadav N., Saha S., Salama E.-S., Li X., Wang L., Jeon B.-H. (2024). Rational Management of the Plant Microbiome for the Second Green Revolution. Plant Commun..

[63] Kandasamy G.D., Kathirvel P. (2023). Insights into Bacterial Endophytic Diversity and Isolation with a Focus on Their Potential Applications –A Review. Microbiol. Res..

[64] Hernández-Tasco A.J., Tronchini R.A., Apaza-Castillo G.A., Hosaka G.K., Quiñones N.R., Goulart M.C., Fantinatti-Garboggini F., Salvador M.J. (2023). Diversity of Bacterial and Fungal Endophytic Communities Presents in the Leaf Blades of *Sinningia magnifica*, *Sinningia schiffneri* and *Sinningia speciosa* from Different Cladus of Gesneriaceae Family: A Comparative Analysis in Three Consecutive Years. Microbiol. Res..

