# Supplementary material for: Stage-Associated Plant–Soil–Microbiome Patterns and Rhizosphere Pb–Zn Stabilization in Indocalamus decorus at a Mining Site
Source: Toxics. 2026 Oct 10;14(10):897. doi: 10.3390/toxics14100897 (PMC13709550; doi:10.3390/toxics14100897)
Supplement: Supplementary file 1 [file toxics-14-00897-s001.zip › toxics-4598650-supplementary.pdf]

Stage-associated plant–soil–microbiome patterns and rhizosphere Pb–Zn stabilization in *Indocalamus decorus* at a mining site

Zhen Miao [1], Fulin Yuan [2], Qiyuan Zou [1], Chenyanan Yuan [1], Yi Zheng [1], Kexin Wang [1], Bingyang Lyu [1], Zhenghua Luo [1], Mingyan Jiang [1] *

[1] *College of Landscape Architecture, Sichuan Agricultural University, Chengdu, Sichuan 611130, China*

[2] *Chengdu Botanical Garden, Chengdu, Sichuan 610083, China*

[*] Corresponding author. E-mail address: jmy@sicau.edu.cn (M. Jiang).

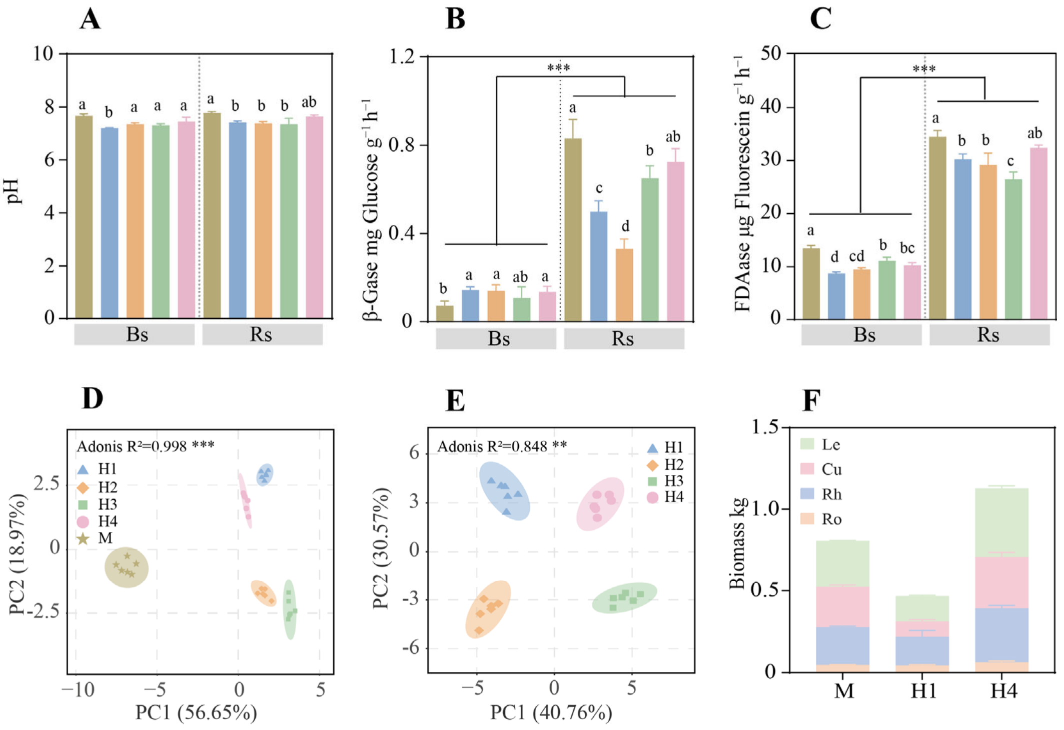

Figure S1 Additional soil measurements, multivariate profiles, and biomass. (A–C) Soil pH, β-glucosidase activity (β-Gase), and fluorescein diacetate hydrolysis activity (FDAase), respectively. (D, E) Principal component analysis (PCA) of nutritional and metal indicators; ellipses show 95% confidence regions. (F) Organ-specific dry biomass per clump. Bars show means ± standard deviation (SD; $n = 6$ for A–E; $n = 3$ for F). Different letters indicate differences among groups within each soil compartment (Tukey's HSD, $p < 0.05$); brackets compare pooled observations between compartments. *, $p < 0.05$; **, $p < 0.01$; ***, $p < 0.001$. M, shoot initiation at the provenance site; H1–H4, shoot initiation, shooting, branch-and-leaf expansion, and rhizome extension at the mining site. Bs, bulk soil; Rs, rhizosphere soil; Ro, roots; Rh, rhizomes; Cu, culms; Le, leaves. Abbreviations and statistical notation apply throughout the supplementary materials.

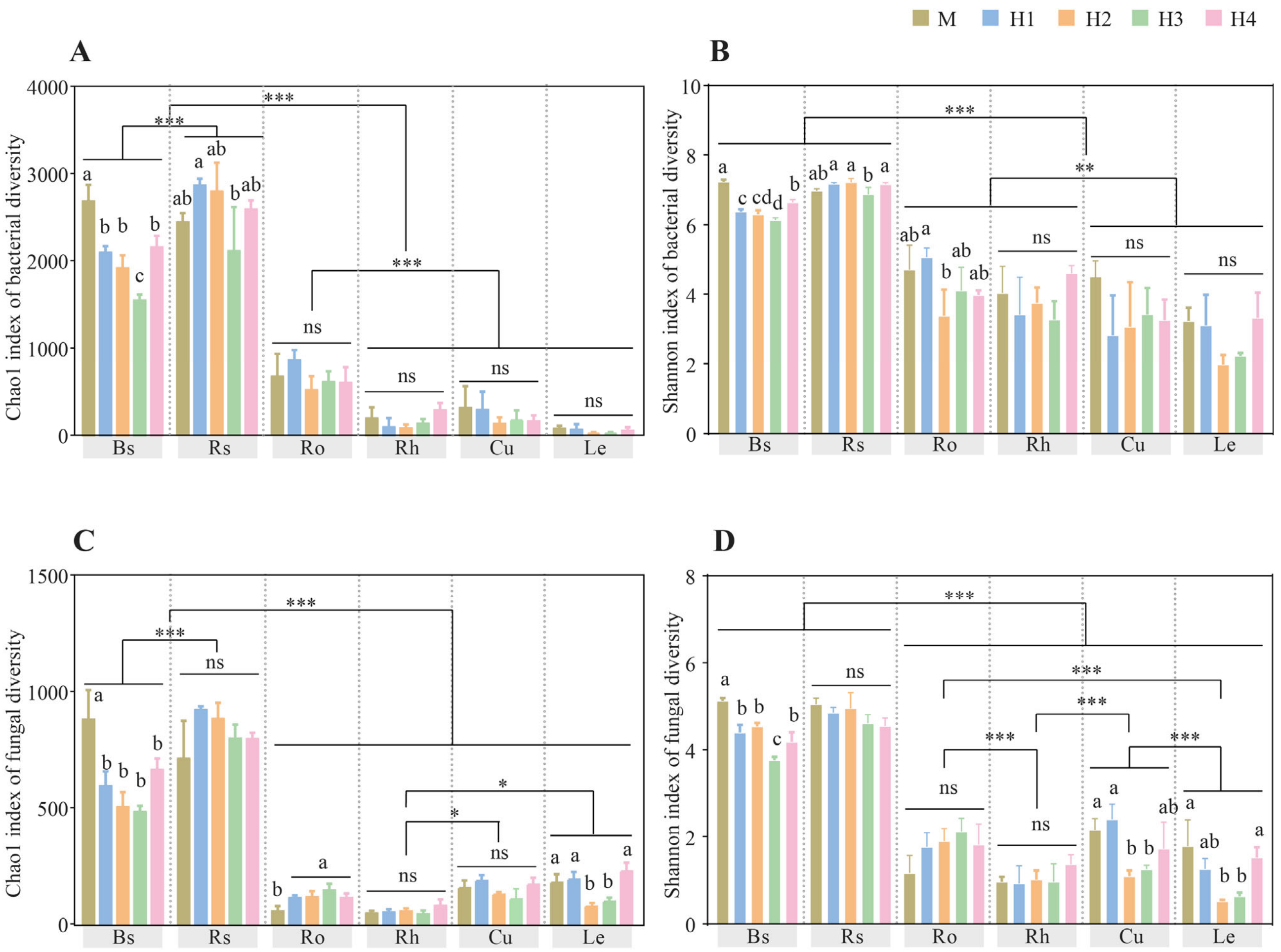

Figure S2 Microbial alpha diversity across compartments and sampling groups. (A, B) Bacterial Chao1 richness and Shannon diversity, respectively. (C, D) Corresponding fungal indices. Bars show means ± SD (n = 3 composite replicates, each combining two individual samples). Letters compare groups within each compartment; brackets compare pooled observations between compartments.

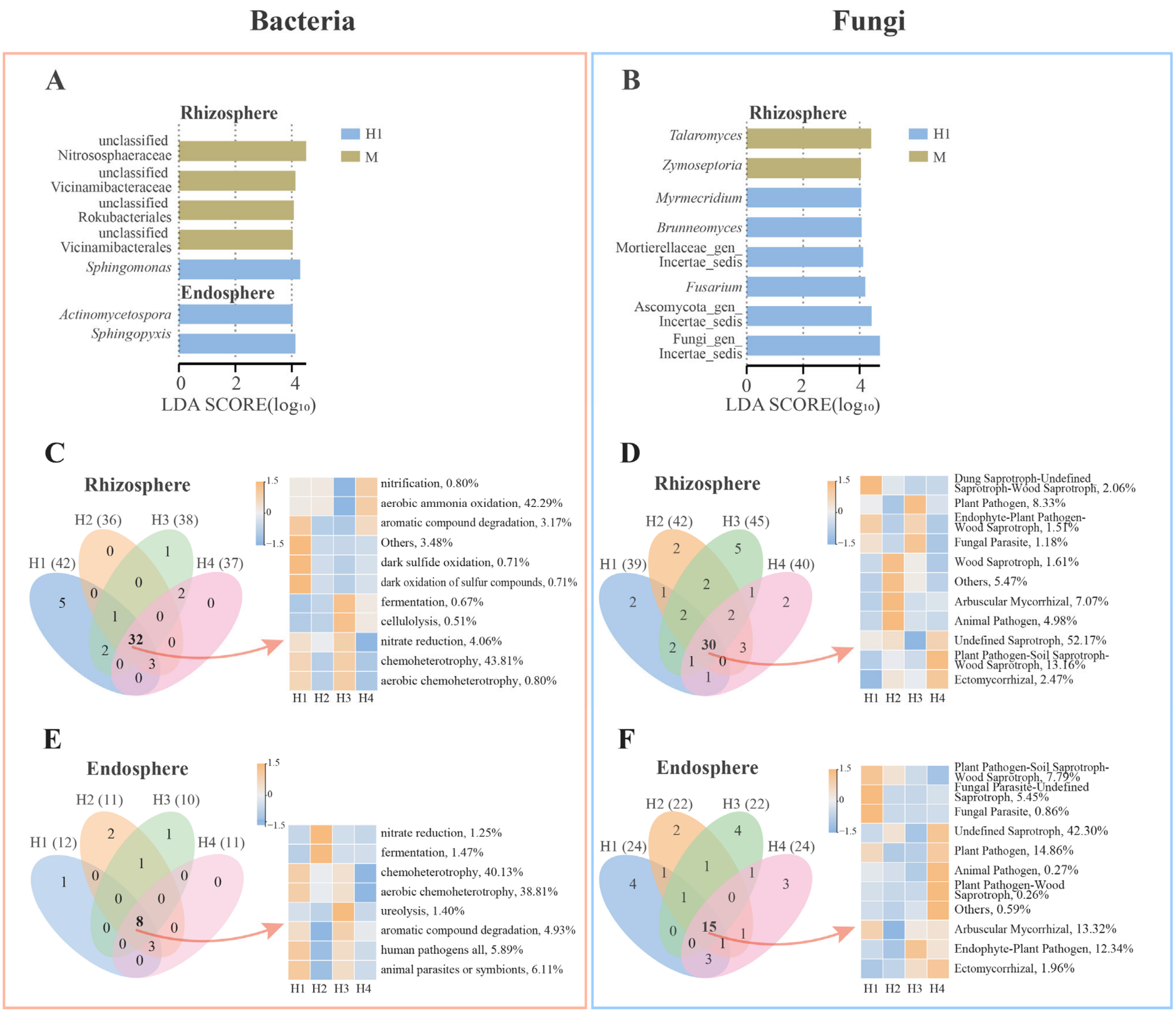

Figure S3 Microbial biomarkers and predicted functional profiles. (A, B) Bacterial and fungal biomarkers between M and H1 identified by linear discriminant analysis effect size (LEfSe; $p < 0.05$; LDA score > 4.0). (C, D) Shared bacterial functions predicted by FAPROTAX and fungal guilds assigned by FUNGuild in the rhizosphere. (E, F) Corresponding endosphere profiles. Venn numbers indicate function or guild counts. Heatmap colours show row-wise Z-scores; warmer colours indicate higher relative abundance within each row. Analyses used three composite replicates per sample type and sampling group. Assignments represent predicted functions, not measured activities.

Table S1 Pb concentrations in BCR fractions and extraction recoveries.

Index	BCR fractionation	Mean content mg kg^{-1}	SD mg kg^{-1}	ΣF1–F4 mg kg^{-1}	Mean of total soil content mg kg^{-1}	Mean recovery %	SD %
Bs1	F1	240.81	23.28	860.63	968.25	88.88	5.98
	F2	196.60	10.11				
	F3	25.65	3.41				
	F4	397.56	24.08				
Bs2	F1	281.37	30.14	1074.21	1128.97	95.19	6.15
	F2	240.03	17.43				
	F3	37.13	8.46				
	F4	515.68	49.65				
Bs3	F1	156.32	9.62	1003.62	1093.95	91.74	2.54
	F2	296.49	8.56				
	F3	20.08	1.18				
	F4	530.74	18.36				
Bs4	F1	140.23	7.84	992.43	1035.78	95.85	3.30
	F2	294.65	7.40				
	F3	17.85	1.95				
	F4	539.70	17.64				
Rs1	F1	67.01	3.74	750.20	787.98	95.19	4.81
	F2	110.01	12.14				
	F3	17.58	0.49				
	F4	555.60	38.34				
Rs2	F1	69.05	5.90	749.15	786.57	95.24	6.23
	F2	111.90	6.10				
	F3	17.47	2.46				
	F4	550.73	42.44				
Rs3	F1	67.41	4.53	861.33	895.73	96.17	4.29
	F2	148.89	5.06				
	F3	23.81	3.72				
	F4	621.22	28.30				
Rs4	F1	57.16	6.81	796.68	819.70	97.18	4.82
	F2	106.67	6.52				
	F3	15.43	2.21				
	F4	617.42	36.05				

Note: Concentrations are expressed on a dry-soil basis (n = 6). F1, acid-soluble/exchangeable; F2, reducible; F3, oxidizable; F4, residual. ΣF1–F4 is the sum of the mean fraction concentrations. For replicate i, recovery (%) = 100 × ($F1_i$ + $F2_i$ + $F3_i$ + $F4_i$)/$C_{soil,i}$, where $C_{soil,i}$ is the independently determined soil concentration. Recovery means and SDs were calculated from the six replicate-level ratios. Bs1–Bs4 and Rs1–Rs4 denote soils at H1–H4. Definitions also apply to Table S2.

Table S2 Zn concentrations in BCR fractions and extraction recoveries.

Index	BCR fractionation	Mean content mg kg^{-1}	SD mg kg^{-1}	ΣF1–F4 mg kg^{-1}	Mean of total soil content mg kg^{-1}	Mean recovery %	SD %
Bs1	F1	388.93	20.04	792.70	855.27	92.69	6.30
	F2	104.34	8.89				
	F3	17.05	2.50				
	F4	282.38	22.49				
Bs2	F1	368.13	13.80	786.82	820.50	95.92	4.86
	F2	123.73	11.73				
	F3	15.58	2.74				
	F4	279.38	26.06				
Bs3	F1	331.60	9.73	766.04	800.88	95.64	4.04
	F2	127.24	3.98				
	F3	14.89	4.22				
	F4	292.30	25.79				
Bs4	F1	289.59	9.93	690.29	807.03	85.52	5.41
	F2	99.53	7.89				
	F3	15.29	0.89				
	F4	285.88	31.34				
Rs1	F1	186.61	7.25	682.05	722.45	94.45	3.69
	F2	101.31	5.30				
	F3	15.00	1.91				
	F4	379.13	15.36				
Rs2	F1	180.86	4.32	643.33	726.08	88.61	4.42
	F2	94.40	10.17				
	F3	14.34	2.79				
	F4	353.74	21.36				
Rs3	F1	195.41	18.10	790.69	851.07	92.90	3.54
	F2	126.07	7.68				
	F3	14.56	1.42				
	F4	454.66	16.22				
Rs4	F1	209.36	12.57	789.77	818.50	96.49	4.69
	F2	138.61	10.32				
	F3	16.51	1.44				
	F4	425.29	23.48				

Table S3 VIP-weighted membership scores of nutritional indicators.

Index	VIP	M		H1		H2		H3		H4	
		mean	SD	mean	SD	mean	SD	mean	SD	mean	SD
TC-Cu	1.700	0.464	0.149	0.456	0.155	0.905	0.280	1.556	0.108	0.300	0.205
AN	1.400	1.328	0.036	0.026	0.028	0.024	0.011	0.442	0.010	0.174	0.054
TC-Le	1.360	1.205	0.116	0.096	0.060	0.671	0.030	0.716	0.062	0.527	0.113
Sucrase	1.340	1.253	0.108	0.173	0.099	0.052	0.027	0.179	0.032	0.084	0.045
Pase	1.340	0.109	0.098	0.861	0.008	1.151	0.064	0.979	0.064	1.075	0.148
TN-Le	1.310	1.283	0.018	0.220	0.041	0.163	0.047	0.027	0.027	0.458	0.049
Urease	1.210	1.160	0.041	0.113	0.004	0.008	0.004	0.110	0.004	0.100	0.003
TP-Ro	1.170	1.022	0.116	0.019	0.017	1.046	0.038	0.498	0.103	0.790	0.160
SOM	1.150	1.075	0.061	0.081	0.043	0.386	0.052	0.164	0.108	0.166	0.021
TP-Rs	1.140	1.129	0.007	0.016	0.009	0.215	0.015	0.062	0.011	0.031	0.008
C Score		1.003	0.025	0.206	0.021	0.462	0.031	0.473	0.024	0.370	0.052

Note: Values are means and SDs of dimensionless weighted scores (n = 6), not original measurements. VIP, variable importance in projection from OPLS-DA. Each membership value was multiplied by its VIP; C score is the mean of the ten weighted indicator scores. TC, total carbon; TN, total nitrogen; TP, total phosphorus; SOM, soil organic matter; AN, available nitrogen; Pase, neutral phosphatase. Suffixes identify compartments; soil indicators refer to rhizosphere soil.

Table S4 VIP-weighted membership scores of metal indicators.

Index	VIP	H1		H2		H3		H4	
		mean	SD	mean	SD	mean	SD	mean	SD
Zn-$TF_{Cu/Ro}$	2.165	1.087	0.148	1.990	0.140	0.253	0.049	0.014	0.012
Zn-$TF_{Le/Cu}$	2.040	0.676	0.096	0.256	0.183	1.160	0.280	1.918	0.098
Zn-BCF_{Cu}	2.032	1.421	0.004	1.907	0.104	0.327	0.006	0.035	0.025
Pb-DTPA	1.709	1.322	0.258	0.610	0.232	0.644	0.188	0.172	0.125
Zn-DTPA	1.495	1.234	0.219	0.861	0.173	0.554	0.371	0.201	0.186
Zn-$TF_{Rh/Ro}$	1.482	1.290	0.114	0.961	0.024	0.103	0.084	0.626	0.029
Zn-F2	1.422	0.398	0.105	0.261	0.202	0.891	0.153	1.140	0.205
Zn-Cu	1.419	0.823	0.082	1.200	0.174	0.388	0.021	0.051	0.036
Pb-BCF_{Rh}	1.362	1.067	0.198	0.629	0.070	0.080	0.069	0.582	0.050
Zn-BCF_{Rh}	1.361	1.329	0.021	0.647	0.005	0.057	0.054	0.607	0.064
Pb-$TF_{Rh/Ro}$	1.156	0.887	0.177	0.506	0.046	0.050	0.051	0.530	0.054
Pb-Rh	1.067	0.815	0.179	0.415	0.061	0.067	0.061	0.437	0.044
HM C Score		1.029	0.053	0.854	0.032	0.381	0.061	0.526	0.028

Note: Score calculation follows Table S3 (n = 6). HM C score is the mean of the twelve weighted scores. BCF, organ/rhizosphere-soil metal concentration ratio; $TF_{x}/_{y}$, organ x/organ y concentration ratio; DTPA, diethylenetriaminepentaacetic acid; F2, reducible fraction. Entries are weighted scores, not original concentrations or ratios.

Table S5 Bacterial amplicon sequencing statistics.

Sample	RawPE	Combined	Qualified	Nochime	Base(nt)	Avglen(nt)	GC	Q20	Q30
MBs11	119448	117803	114935	48782	20437437	418.95	58.45%	98.67%	95.36%
MBs12	103237	101740	99310	43383	18140005	418.14	58.40%	98.67%	95.36%
MBs13	104329	103002	100837	44987	18769449	417.22	58.41%	98.76%	95.64%
MRs11	105988	104526	102092	46762	19522861	417.49	57.21%	98.70%	95.42%
MRs12	104408	103034	100924	44189	18461003	417.77	57.23%	98.65%	95.37%
MRs13	115425	113896	111347	47142	19711329	418.13	57.27%	98.70%	95.50%
MRo11	112577	111657	109235	99140	40570150	409.22	55.56%	98.85%	95.82%
MRo12	105415	104256	101561	91018	37142895	408.08	55.27%	98.79%	95.63%
MRo13	104842	103916	101033	90384	36877714	408.01	55.55%	98.76%	95.42%
MRh11	106330	105522	103081	102454	41623741	406.27	55.07%	98.85%	95.73%
MRh12	105077	104017	101369	100676	40877210	406.03	55.19%	98.79%	95.53%
MRh13	102925	102025	99425	91273	37154729	407.07	54.81%	98.88%	95.89%
MCu11	103211	102449	100227	99065	40244005	406.24	55.19%	98.91%	95.95%
MCu12	103959	103157	100650	92517	37816258	408.75	56.37%	98.82%	95.76%
MCu13	106609	105485	102949	102083	41481711	406.35	55.26%	98.82%	95.66%
MLe11	102269	101399	98864	98471	39983436	406.04	55.12%	98.83%	95.76%
MLe12	104061	103077	100208	99379	40349449	406.02	55.15%	98.72%	95.41%
MLe13	95846	95177	92774	92394	37513688	406.02	54.97%	98.91%	96.00%
HBs11	103827	102311	99549	71484	29927418	418.66	56.15%	98.61%	95.07%
HBs12	106766	105211	102422	73598	30756063	417.89	56.09%	98.50%	94.80%
HBs13	103132	101741	99143	77304	32204509	416.6	56.12%	98.54%	94.97%
HRs11	103445	101827	99343	72060	29975074	415.97	56.57%	98.55%	94.97%
HRs12	102492	100667	98062	73231	30419829	415.4	56.41%	98.53%	94.89%
HRs13	102531	100874	98317	72181	30062234	416.48	56.59%	98.60%	95.10%
HRo11	117750	117097	114022	107282	44156205	411.59	55.41%	98.60%	95.06%
HRo12	102352	101591	98475	96171	39213871	407.75	55.48%	98.51%	94.85%
HRo13	103467	102774	99921	97690	39799899	407.41	55.28%	98.57%	94.87%
HRh11	105302	104708	101815	101519	41217380	406.01	55.02%	98.63%	94.97%
HRh12	118339	117542	114344	114103	46331272	406.05	55.11%	98.65%	95.16%
HRh13	103506	102805	99740	98649	40105427	406.55	55.27%	98.53%	94.59%
HCu11	105739	104874	102531	96550	39862703	412.87	55.06%	98.90%	95.98%
HCu12	102827	101880	99470	96312	39248798	407.52	56.03%	98.87%	95.84%
HCu13	109795	108800	106241	96246	39192067	407.21	55.71%	98.83%	95.64%
HLe11	105968	105334	102479	102013	41433575	406.16	55.22%	98.65%	94.99%
HLe12	56899	56541	54909	54404	22087450	405.99	55.15%	98.75%	95.50%
HLe13	102654	101909	98686	98232	39890225	406.08	55.19%	98.38%	94.07%
HBs21	103534	101526	99609	67622	28280924	418.22	56.41%	98.82%	95.68%
HBs22	102045	100361	98478	73375	30663465	417.9	56.44%	98.81%	95.74%
HBs23	104438	102742	100757	72376	30260388	418.1	56.44%	98.80%	95.70%
HRs21	110754	108386	106202	65409	27157861	415.2	57.00%	98.83%	95.77%
HRs22	106530	104234	102425	78305	32579506	416.06	56.95%	98.85%	95.83%
HRs23	94486	92688	91068	58218	24175915	415.27	57.07%	98.82%	95.78%
HRo21	119067	118481	116091	104487	42812006	409.74	56.22%	98.79%	95.55%
HRo22	119105	118637	116529	111368	45373920	407.42	55.88%	98.85%	95.79%
HRo23	102538	102113	100889	94265	38666889	410.19	57.17%	98.90%	96.06%
HRh21	105045	104446	102517	101961	41400853	406.05	55.27%	98.87%	95.76%
HRh22	106263	105734	103783	103220	41911344	406.04	55.19%	98.92%	95.87%
HRh23	102040	101510	99472	98941	40172471	406.02	55.17%	98.89%	95.82%
HCu21	102758	102187	100033	99425	40373929	406.07	55.19%	98.88%	95.69%
HCu22	104920	104469	102560	101247	41226413	407.19	56.11%	98.90%	95.95%
HCu23	103517	102970	100890	100387	40764355	406.07	55.17%	98.80%	95.59%
HLe21	103995	103481	101506	101104	41049726	406.01	55.07%	98.82%	95.57%
HLe22	104973	104467	102323	101805	41334023	406.01	55.12%	98.88%	95.80%
HLe23	104376	103827	101678	101257	41109440	405.99	55.15%	98.87%	95.72%

Table S5 (cont.)

Sample	RawPE	Combined	Qualified	Nochime	Base(nt)	Avglen(nt)	GC	Q20	Q30
HBs31	102772	101696	99791	56833	23553561	414.43	56.59%	98.85%	95.86%
HBs32	94583	93647	91987	63175	26199154	414.71	56.63%	98.87%	95.98%
HBs33	115288	114081	112177	64134	26587712	414.57	56.59%	98.90%	95.99%
HRs31	103407	102024	99852	54141	22466789	414.97	56.83%	98.76%	95.54%
HRs32	104044	102765	100954	61130	25396893	415.46	56.74%	98.86%	95.85%
HRs33	50577	50243	49418	30380	12609057	415.04	56.78%	98.91%	96.09%
HRo31	106789	106348	105322	95454	38977375	408.34	56.33%	98.94%	96.15%
HRo32	106111	105633	103554	92715	38169246	411.68	55.98%	98.84%	95.73%
HRo33	110481	109969	107880	102955	42042360	408.36	55.76%	98.86%	95.68%
HRh31	112053	111348	109187	108546	44079993	406.1	55.27%	98.83%	95.57%
HRh32	106184	105569	103485	102365	41662905	407	55.14%	98.89%	95.77%
HRh33	102998	102335	100504	99691	40483561	406.09	55.28%	98.96%	95.98%
HCu31	98167	97707	95960	94862	38535718	406.23	55.50%	98.88%	95.79%
HCu32	102637	102213	100227	96290	39261606	407.74	55.84%	98.94%	96.02%
HCu33	102179	101655	99530	99001	40209474	406.15	55.19%	98.87%	95.75%
HLe31	102573	102205	101129	100543	40820641	406	55.13%	99.06%	96.21%
HLe32	102472	102019	100732	100328	40732261	405.99	55.08%	98.89%	95.72%
HLe33	103921	103424	101216	100833	40938807	406.01	55.07%	98.85%	95.58%
HBs41	102582	101633	98458	61451	25633580	417.14	56.54%	98.54%	95.01%
HBs42	104733	103653	99683	71557	29824896	416.8	56.65%	98.40%	94.56%
HBs43	104552	103514	99898	69115	28833015	417.17	56.71%	98.51%	94.87%
HRs41	102022	100819	97846	55513	23101052	416.14	56.99%	98.61%	95.13%
HRs42	106350	103810	100202	56603	23545145	415.97	56.91%	98.48%	94.84%
HRs43	105108	103753	100095	53243	22159057	416.19	57.05%	98.46%	94.72%
HRo41	103562	102803	99087	92215	37683851	408.65	56.50%	98.56%	94.98%
HRo42	112547	111580	107712	100056	40817617	407.95	56.06%	98.55%	94.96%
HRo43	102730	101963	98505	92541	37652602	406.87	55.22%	98.50%	94.79%
HRh41	104369	103552	100040	98901	40231327	406.78	55.19%	98.53%	94.77%
HRh42	106299	105528	102109	101576	41291451	406.51	54.97%	98.57%	94.91%
HRh43	119765	118755	114894	113265	46053559	406.6	55.06%	98.59%	95.03%
HCu41	110419	109336	105146	104552	42502108	406.52	55.18%	98.43%	94.36%
HCu42	104515	103595	100051	99295	40350927	406.37	55.16%	98.54%	94.75%
HCu43	104198	103097	99857	99441	40479446	407.07	55.44%	98.65%	95.31%
HLe41	102905	101866	98062	97718	39682989	406.1	55.20%	98.40%	94.24%
HLe42	105089	104164	100347	100038	40622778	406.07	55.08%	98.50%	94.73%
HLe43	109270	108321	104496	104145	42283071	406	55.09%	98.49%	94.62%

Note: RawPE, raw paired-end reads; Combined, merged reads; Qualified, quality-filtered reads; Nochime, non-chimeric reads; Base, total nucleotides; Avglen, mean read length; nt, nucleotides; GC, guanine–cytosine percentage; Q20/Q30, percentages of bases with Phred scores ≥20/≥30. Sample identifiers specify site, compartment, stage, and composite replicate; for example, HBs31 denotes Site H bulk soil at H3, replicate 1. Definitions also apply to Table S6.

Table S6 Fungal amplicon sequencing statistics.

Sample	RawPE	Combined	Qualified	Nochime	Base(nt)	Avglen(nt)	GC	Q20	Q30
MBs11	105177	103246	103069	99107	24478639	246.99	48.03%	99.59%	98.26%
MBs12	105502	103597	103314	99347	24970311	251.34	48.45%	99.51%	97.90%
MBs13	102117	100041	99742	95926	24247228	252.77	46.76%	99.51%	97.97%
MRs11	118094	115889	115665	109783	26892627	244.96	49.44%	99.55%	98.07%
MRs12	106061	104199	103951	99590	24897131	250	48.59%	99.52%	97.96%
MRs13	106373	102380	102141	97772	24310358	248.64	49.12%	99.52%	97.95%
MRo11	65009	64769	64554	64541	16337049	253.13	54.69%	98.87%	96.42%
MRo12	105513	104955	104618	104489	26429189	252.94	53.87%	99.03%	96.88%
MRo13	105223	104011	103098	102967	28846424	280.15	61.89%	97.41%	92.38%
MRh11	102002	101467	100981	100806	26183301	259.74	56.88%	98.36%	95.11%
MRh12	103891	103197	102012	101985	29419519	288.47	64.67%	97.05%	91.27%
MRh13	111091	110118	108970	108959	32130675	294.89	66.24%	96.71%	90.49%
MCu11	116762	115424	114663	112047	29875358	266.63	55.97%	98.09%	94.12%
MCu12	106657	105100	104558	103119	27279710	264.55	56.44%	98.35%	94.92%
MCu13	107151	106290	105662	104365	28566601	273.72	58.81%	98.07%	94.04%
MLe11	105270	103236	102616	101801	26938491	264.62	56.85%	98.15%	94.27%
MLe12	78810	77945	77358	77135	21594345	279.96	63.17%	97.57%	92.68%
MLe13	103923	102386	101630	101024	28924945	286.32	64.73%	97.32%	92.03%
HBs11	105616	103408	103158	93608	22637412	241.83	47.89%	99.52%	98.03%
HBs12	105195	102190	101866	92948	22371420	240.69	48.51%	99.43%	97.75%
HBs13	103099	100407	100184	93470	22338872	239	48.23%	99.59%	98.25%
HRs11	105924	102710	102335	93040	23275414	250.17	48.05%	99.43%	97.65%
HRs12	115928	114528	114283	107487	26157088	243.35	49.02%	99.49%	97.89%
HRs13	103812	102263	101986	95886	23857576	248.81	48.48%	99.43%	97.67%
HRo11	117700	116661	115191	114863	33252622	289.5	64.83%	97.30%	91.96%
HRo12	117952	116619	115648	115229	30123678	261.42	59.12%	98.18%	94.21%
HRo13	105220	104150	102943	102247	28931272	282.95	63.10%	97.54%	92.57%
HRh11	106198	105382	104163	104150	30799763	295.73	66.45%	97.33%	91.63%
HRh12	104611	103781	102531	102518	30281470	295.38	66.34%	97.37%	92.13%
HRh13	102056	101297	100206	100018	28029431	280.24	62.62%	97.71%	93.05%
HCu11	105824	104634	103962	102020	26933549	264	58.80%	98.10%	94.23%
HCu12	103422	102235	101311	100170	27498618	274.52	61.40%	97.38%	92.30%
HCu13	119658	117899	117207	114946	29646412	257.92	56.34%	98.10%	94.47%
HLe11	103057	101660	100340	100056	28979075	289.63	65.27%	97.37%	91.89%
HLe12	102143	100988	99929	99118	27626481	278.72	59.83%	97.72%	92.45%
HLe13	109505	107982	106683	106265	30545101	287.44	64.61%	97.45%	91.98%
HBs21	103710	101755	101558	93959	22281498	237.14	48.60%	99.58%	98.22%
HBs22	104485	102212	101990	93064	22498390	241.75	48.25%	99.52%	97.99%
HBs23	90940	89172	88989	82575	19906931	241.08	48.09%	99.56%	98.12%
HRs21	104814	103005	102676	96581	24015128	248.65	50.54%	99.28%	97.24%
HRs22	108468	105886	105539	100017	24849350	248.45	48.93%	99.39%	97.51%
HRs23	106721	105331	104871	99597	26455477	265.63	48.32%	99.30%	97.22%
HRo21	91720	90729	90002	89598	25026535	279.32	63.34%	97.86%	93.10%
HRo22	97435	96356	95597	94923	26680088	281.07	63.67%	97.97%	93.54%
HRo23	102096	100507	100046	93928	24920419	265.31	59.85%	98.60%	95.39%
HRh21	102681	102047	100898	100878	29754577	294.96	66.33%	97.64%	92.49%
HRh22	102718	102243	101253	101237	29818915	294.55	65.89%	97.79%	92.80%
HRh23	105661	105000	103875	103725	30444983	293.52	63.88%	97.66%	92.74%
HCu21	103883	103080	101970	101876	29815503	292.66	65.75%	97.69%	92.74%
HCu22	107870	107173	105937	105887	31135750	294.05	66.14%	97.66%	92.48%
HCu23	103609	102845	101879	101800	29646744	291.23	65.49%	97.84%	92.89%
HLe21	117897	116929	115878	115803	34077047	294.27	66.32%	97.58%	92.54%
HLe22	108168	107275	106269	106211	31247620	294.2	66.31%	97.40%	92.02%
HLe23	106497	105760	104664	104615	30831650	294.72	66.42%	97.44%	92.12%

Table S6 (cont.)

Sample	RawPE	Combined	Qualified	Nochime	Base(nt)	Avglen(nt)	GC	Q20	Q30
HBs31	105457	104019	103841	93631	22035853	235.35	50.04%	99.61%	98.25%
HBs32	106479	104804	104547	94698	22629968	238.97	49.83%	99.52%	97.94%
HBs33	104591	102865	102702	95117	22440965	235.93	49.86%	99.59%	98.20%
HRs31	106364	104585	104317	96653	24381136	252.25	48.27%	99.48%	97.79%
HRs32	103618	101071	100754	95534	23271614	243.6	48.78%	99.34%	97.45%
HRs33	103814	101870	101457	95296	24193186	253.87	48.40%	99.33%	97.31%
HRo31	119758	118718	118113	116761	30610621	262.16	56.35%	98.69%	95.61%
HRo32	105026	101392	100316	97739	27656475	282.96	61.79%	97.12%	91.35%
HRo33	109305	106978	106551	106112	27841797	262.38	56.76%	98.84%	96.10%
HRh31	105559	105020	104008	103988	30753323	295.74	66.50%	97.48%	92.18%
HRh32	145057	138178	136380	135945	40041833	294.54	66.34%	96.00%	88.82%
HRh33	108072	107464	106598	106493	29745133	279.32	61.39%	98.06%	93.90%
HCu31	102482	101446	100522	100477	29139551	290.01	65.14%	97.81%	92.78%
HCu32	105598	104639	103769	100260	28771868	286.97	64.15%	97.90%	93.25%
HCu33	105333	104110	103119	103021	29485457	286.21	64.22%	97.76%	92.73%
HLe31	106440	105588	104694	104528	30692779	293.63	66.21%	97.64%	92.70%
HLe32	104776	103856	102493	102377	30138198	294.38	66.27%	97.33%	91.44%
HLe33	106605	105197	104153	103938	30209257	290.65	65.81%	97.52%	92.36%
HBs41	102265	100688	100378	96292	24278509	252.13	48.90%	99.49%	97.95%
HBs42	105947	104376	104072	100037	25131324	251.22	49.21%	99.52%	98.03%
HBs43	105763	104170	103696	100256	26073818	260.07	48.85%	99.43%	97.71%
HRs41	106648	103963	103631	94861	23241667	245.01	48.84%	99.50%	97.93%
HRs42	102985	100867	100566	93435	22940567	245.52	49.24%	99.54%	98.05%
HRs43	102149	99956	99629	94063	23228068	246.94	49.26%	99.49%	97.90%
HRo41	102654	101426	100199	89241	25397146	284.59	63.64%	97.44%	92.36%
HRo42	107626	107039	106565	103308	26244903	254.05	54.87%	98.97%	96.61%
HRo43	105279	96177	95225	90980	22428012	246.52	53.79%	97.40%	93.49%
HRh41	139212	137270	135895	116472	33923872	291.26	65.48%	97.43%	91.87%
HRh42	113964	113036	111997	100960	28681403	284.09	63.72%	97.75%	93.17%
HRh43	102099	101599	100678	92753	25040041	269.96	60.04%	98.22%	94.49%
HCu41	118748	116961	115527	100517	28487550	283.41	64.21%	97.52%	92.50%
HCu42	104341	102980	102103	86821	23321332	268.61	59.74%	98.11%	94.25%
HCu43	106000	104819	103253	91359	26716207	292.43	65.70%	97.04%	91.32%
HLe41	104329	102321	101195	91457	25444328	278.21	63.05%	97.62%	92.85%
HLe42	106578	104516	103381	92569	25491194	275.38	62.56%	97.74%	93.22%
HLe43	106515	104541	103122	92088	26046816	282.85	64.28%	97.47%	92.43%

Table S7 Top 20 rhizosphere bacterial genera enriched at H1 relative to M, ranked by VIP.

Genus	VIP	P value	Fold Change
Streptomyces	1.92	3.72E-08	18.36
Iamia	1.83	1.24E-07	6.59
Kribbella	1.83	8.00E-08	21.30
Unclassified Acidimicrobiaceae	1.82	1.32E-07	8.95
Nocardioides	1.80	1.28E-07	11.84
Variovorax	1.80	6.99E-07	12.66
Pseudomonas	1.80	1.46E-08	4.64
Unclassified 67-14	1.79	2.46E-07	7.07
Acidiferrimicrobium	1.78	2.92E-07	3.36
Unclassified Nevskiaceae	1.78	6.34E-06	9.64
Pseudonocardia	1.77	4.69E-07	11.06
Lentzea	1.76	6.87E-07	43.04
Phenylobacterium	1.76	4.03E-07	5.20
Virgisporangium	1.75	3.77E-07	25.25
Microbacterium	1.75	3.95E-07	4.75
Unclassified Longimicrobiaceae	1.73	5.19E-07	6.13
Sphingomonas	1.70	9.34E-07	4.44
Massilia	1.69	2.26E-06	9.80
Chryseobacterium	1.22	3.37E-08	5.33
Bacillus	1.22	2.86E-07	6.86

Note: Exploratory screening used Welch's $p < 0.05$, VIP > 1, and fold change (FC; H1/M) > 2. Bold type indicates genera enriched in both rhizosphere and endosphere screening results. Infinite FC indicates zero mean abundance at M and positive abundance at H1. Criteria and notation apply to Tables S8–S10.

Table S8 Top 20 rhizosphere fungal genera enriched at H1 relative to M, ranked by VIP.

Genus	VIP	P value	Fold Change
Penicillium	2.24	1.15E-07	6.18
Pleosporales_gen_Incertae_sedis	1.93	4.38E-06	23.71
Fungi_gen_Incertae_sedis	1.87	1.28E-05	6.81
Fusarium	1.86	4.32E-05	3.16
Dothideomycetes_gen_Incertae_sedis	1.73	3.90E-05	161.68
Plectosphaerellaceae_gen_Incertae_sedis	1.63	8.86E-05	19.80
Achroiostachys	1.56	2.26E-04	89.37
Sarocladium	1.49	3.17E-04	12.00
Montagnula	1.48	4.92E-04	11.19
Macrophomina	1.47	1.66E-04	inf
Parachaetomium	1.47	5.06E-04	181.17
Albifimbria	1.45	3.70E-03	3.70
Ceratobasidium	1.39	1.94E-03	4.65
Herpotrichiellaceae_gen_Incertae_sedis	1.38	8.49E-04	14.96
Lulworthiaceae_gen_Incertae_sedis	1.38	7.63E-04	24.11
**Agaricales_gen_Incertae_sedis**	1.32	3.66E-03	3.06
Didymocyrtis	1.27	1.01E-02	6.13
Myrmecridium	1.24	2.54E-03	8.93
Mortierellaceae_gen_Incertae_sedis	1.23	1.81E-02	2.37
Orbilia	1.22	2.57E-03	inf

Table S9 Endosphere bacterial genera enriched at H1 relative to M, ranked by VIP.

Genus	VIP	P value	Fold Change
Streptomyces	1.67	2.24E-04	2.15
Nordella	1.35	2.37E-03	6.21
Staphylococcus	1.31	4.44E-02	4.23
Inquilinus	1.28	2.33E-02	inf
Chryseobacterium	1.27	2.07E-03	9.04
Kribbella	1.21	1.02E-02	7.25
Phyllobacterium	1.18	5.21E-03	inf
Catelliglobosispora	1.16	1.53E-02	5.77
Rubellimicrobium	1.16	6.75E-03	5.63
Pseudomonas	1.14	2.49E-02	5.40
Luteitalea	1.12	2.14E-02	6.13
Rubrobacter	1.10	2.44E-02	inf
MM2	1.10	1.98E-02	inf
Gemmata	1.10	4.43E-02	inf
Bacillus	1.08	4.90E-02	12.81
Sphingobacterium	1.08	1.24E-02	inf
**Unclassified LWQ8**	1.07	2.60E-02	10.26
Mycobacterium	1.07	2.52E-02	3.96
Paracoccus	1.07	1.63E-02	inf
Blastococcus	1.07	9.18E-03	15.20
Lentzea	1.05	2.76E-02	7.98
Amycolatopsis	1.04	2.20E-02	97.50
Enterobacter	1.03	3.66E-02	inf
Undibacterium	1.02	1.59E-02	inf
Lacibacter	1.00	4.74E-02	4.17

Table S10 Endosphere fungal genera enriched at H1 relative to M, ranked by VIP.

Genus	VIP	P value	Fold Change
Fusarium	1.67	2.27E-04	6.58
Lecanicillium	1.09	6.82E-03	2.17
Dioszegia	1.06	3.40E-03	21.74
**Agaricales_gen_Incertae_sedis**	1.04	2.24E-03	4.92
Penicillium	1.02	3.36E-02	2.20
